# Isolation of an archaeon at the prokaryote–eukaryote interface

**DOI:** 10.1038/s41586-019-1916-6

**Published:** 2020-01-15

**Authors:** Hiroyuki Imachi, Masaru K. Nobu, Nozomi Nakahara, Yuki Morono, Miyuki Ogawara, Yoshihiro Takaki, Yoshinori Takano, Katsuyuki Uematsu, Tetsuro Ikuta, Motoo Ito, Yohei Matsui, Masayuki Miyazaki, Kazuyoshi Murata, Yumi Saito, Sanae Sakai, Chihong Song, Eiji Tasumi, Yuko Yamanaka, Takashi Yamaguchi, Yoichi Kamagata, Hideyuki Tamaki, Ken Takai

**Affiliations:** 1 Institute for Extra-cutting-edge Science and Technology Avant-garde Research (X-star), Japan Agency for Marine-Earth Science and Technology (JAMSTEC), Yokosuka, Japan; 2 Bioproduction Research Institute, National Institute of Advanced Industrial Science and Technology (AIST), Tsukuba, Japan; 3 Department of Civil and Environmental Engineering, Nagaoka University of Technology, Nagaoka, Japan; 4 Kochi Institute for Core Sample Research, X-star, JAMSTEC, Nankoku, Japan; 5 Biogeochemistry Program, Research Institute for Marine Resources Utilization, JAMSTEC, Yokosuka, Japan; 6 Department of Marine and Earth Sciences, Marine Work Japan, Yokosuka, Japan; 7 Research Institute for Global Change, JAMSTEC, Yokosuka, Japan; 8 Research Institute for Marine Resources Utilization, JAMSTEC, Yokosuka, Japan; 9 National Institute for Physiological Sciences, Okazaki, Japan; 10 Section for Exploration of Life in Extreme Environments, Exploratory Research Center on Life and Living Systems (ExCELLS), National Institute of Natural Sciences, Okazaki, Japan

**Keywords:** Archaeal biology, Archaeal evolution

## Abstract

The origin of eukaryotes remains unclear^1–4^. Current data suggest that eukaryotes may have emerged from an archaeal lineage known as ‘Asgard’ archaea^5,6^. Despite the eukaryote-like genomic features that are found in these archaea, the evolutionary transition from archaea to eukaryotes remains unclear, owing to the lack of cultured representatives and corresponding physiological insights. Here we report the decade-long isolation of an Asgard archaeon related to Lokiarchaeota from deep marine sediment. The archaeon—‘*Candidatus* Prometheoarchaeum syntrophicum’ strain MK-D1—is an anaerobic, extremely slow-growing, small coccus (around 550 nm in diameter) that degrades amino acids through syntrophy. Although eukaryote-like intracellular complexes have been proposed for Asgard archaea^6^, the isolate has no visible organelle-like structure. Instead, *Ca*. P. syntrophicum is morphologically complex and has unique protrusions that are long and often branching. On the basis of the available data obtained from cultivation and genomics, and reasoned interpretations of the existing literature, we propose a hypothetical model for eukaryogenesis, termed the entangle–engulf–endogenize (also known as E^3^) model.

## Abstract (web summary)

Isolation and characterization of an archaeon that is most closely related to eukaryotes reveals insights into how eukaryotes may have evolved from prokaryotes.

## Main

How the first eukaryotic cell emerged remains unclear. Among various competing evolutionary models, the most widely accepted are symbiogenic models in which an archaeal host cell and an alphaproteobacterial endosymbiont merged to become the first eukaryotic cell^1–4^. Recent metagenomic characterization of deep-sea archaeal group/marine benthic group-B (also known as Lokiarchaeota) and the Asgard archaea superphylum led to the theory that eukaryotes originated from an archaeon that was closely related to these lineages^5,6^. The genomes of Asgard archaea encode a repertoire of proteins that are only found in Eukarya (eukaryotic signature proteins), including those involved in membrane trafficking, vesicle formation and/or transportation, ubiquitin and cytoskeleton formation^6^. Subsequent metagenomic studies have suggested that Asgard archaea have a wide variety of physiological properties, including hydrogen-dependent anaerobic autotrophy^7^, peptide or short-chain hydrocarbon-dependent organotrophy^8–12^ and rhodopsin-based phototrophy^13,14^. However, no representative of the Asgard archaea has been cultivated and, thus, the physiology and cell biology of this clade remains unclear. In an effort to close this knowledge gap, we successfully isolated an archaeon of this clade, report its physiological and genomic characteristics, and propose a new model for eukaryogenesis.

## Isolation of an Asgard archaeon

**Fig. 1:**
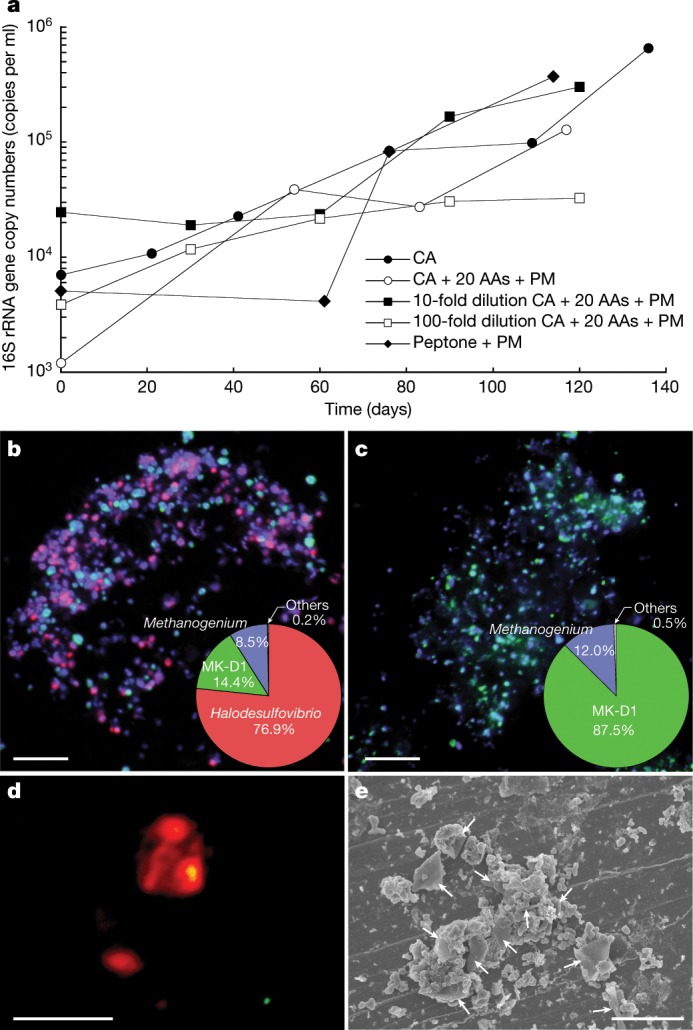
Growth curves and photomicrographs of the cultured Lokiarchaeota strain MK-D1. **a**, Growth curves of MK-D1 in anaerobic medium supplemented with casamino acids (CA) alone; casamino acids with 20 amino acids (AAs) and powdered milk (PM); or peptone with powdered milk. Results are also shown for cultures fed with 10- and 100-fold dilution of casamino acids, 20 amino acids and powdered milk. **b**, **c**, Fluorescence images of cells from enrichment cultures after 8 (**b**) and 11 (**c**) transfers stained with DAPI (violet) and hybridized with nucleotide probes that target MK-D1 (green) and Bacteria (red). Pie charts show the relative abundance of microbial populations based on SSU rRNA gene-tag sequencing (iTAG) analysis. **d**, A fluorescence image of cells from enrichment cultures after 11 transfers hybridized with nucleotide probes that target MK-D1 (green) and *Methanogenium* (red). The FISH experiments were performed three times with similar results. **e**, SEM image of a highly purified co-culture of MK-D1 and *Methanogenium*. White arrows indicate *Methanogenium* cells. We observed four different co-cultures with *Methanogenium*. Representative of *n* = 40 recorded images. The detailed iTAG-based community compositions of cultures corresponding to each of the images are shown in Supplementary Table 1. Scale bars, 10 μm (**b**, **c**) and 5 μm (**d**, **e**).

**Extended Data Fig. 1:**
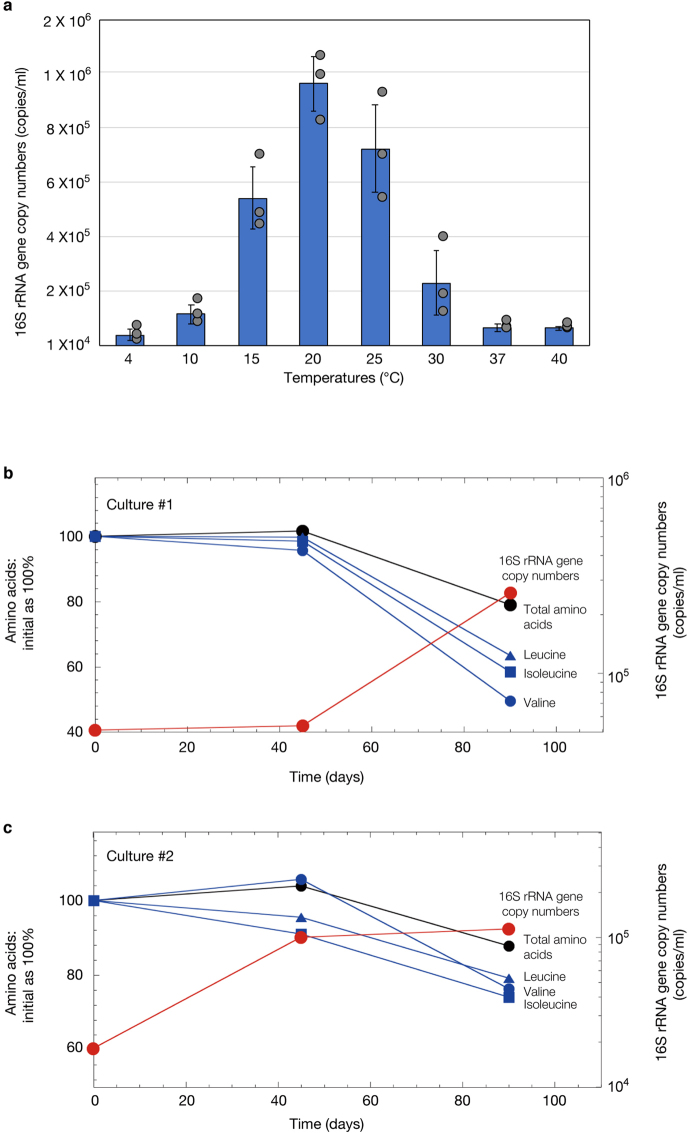
Growth of MK-D1. **a**, Effect of temperature on growth of MK-D1. Data are mean ± s.d. of triplicate determinations. Each data point is shown as a dot. The temperature range test was performed twice with similar results. **b**, **c**, The amino acid concentrations and growth curves of MK-D1 in pure cocultures at 20 °C. Results from cultures 1 (**b**) and 2 (**c**) are shown. Please note that the initial concentrations of amino acids were normalized to 100%. Total amino acids and several representative amino acids (Val, valine; Leu, leucine; Ile, isoleucine) are independently shown for the duplicate culture samples. Detailed iTAG-based community compositions of the cultures are shown in Supplementary Table 1.

Setting out to isolate uncultivated deep marine sediment microorganisms, we engineered and operated a methane-fed continuous-flow bioreactor system for more than 2,000 days to enrich such organisms from anaerobic marine methane-seep sediments^15^ (Supplementary Note 1). We successfully enriched many phylogenetically diverse yet-to-be cultured microorganisms, including Asgard archaea members (Loki-, Heimdall- and Odinarchaeota)^15^. For further enrichment and isolation, samples of the bioreactor community were inoculated in glass tubes with simple substrates and basal medium. After approximately one year, we found faint cell turbidity in a culture containing casamino acids supplemented with four bacteria-suppressing antibiotics (Supplementary Note 2) that was incubated at 20 °C. Clone library-based small subunit (SSU) rRNA gene analysis revealed a simple community that contained *Halodesulfovibrio* and a small population of Lokiarchaeota (Extended Data Table 1). In pursuit of this archaeon, which we designated strain MK-D1, we repeated subcultures when MK-D1 reached maximum cell densities as measured by quantitative PCR (qPCR). This approach gradually enriched the archaeon, which has an extremely slow growth rate and low cell yield (Fig. 1a). The culture consistently had a 30–60-day lag phase and required more than 3 months to reach full growth: around 10^5^ 16S rRNA gene copies ml^−1^ (Fig. 1a). The doubling time was estimated to be approximately 14–25 days. Variation in cultivation temperatures (Extended Data Fig. 1), and substrate combinations and concentrations did not significantly shorten the lag phase or improve growth rate or cell yield (data not shown). Static cultivation supplemented with 20 amino acids and powdered milk resulted in the stable growth. For further characterization, we cultured the archaeon under the optimal conditions determined above.

**Fig. 2:**
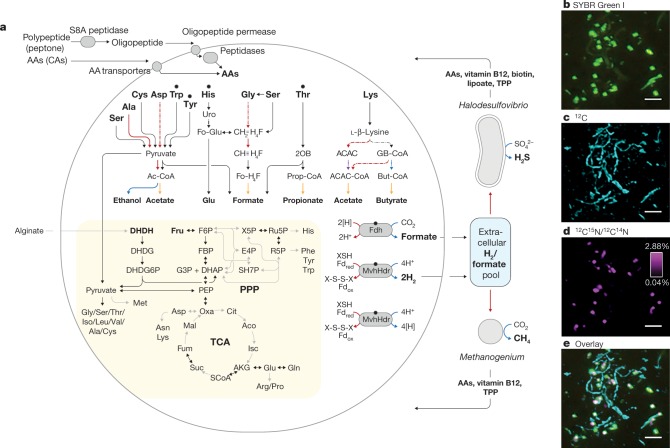
Syntrophic amino acid utilization of MK-D1. **a**, Genome-based metabolic reconstruction of MK-D1. Metabolic pathways identified (coloured or black) and not identified (grey) are shown. For identified pathways, each step (solid line) or process (dotted) is marked by whether it is oxidative (red), reductive (blue), ATP-yielding (orange) or ATP-consuming (purple). Wavy arrows indicate exchange of compounds: formate, H_2_, amino acids, vitamin B_12_, biotin, lipoate and thiamine pyrophosphate (TPP), which are predicted to be metabolized or synthesized by the partnering *Halodesulfovibrio* and/or *Methanogenium*. Biosynthetic pathways are indicated with a yellow background. Metatranscriptomics-detected amino-acid-catabolizing pathways are indicated (black dots above amino acids). DHDH, 4,5-dihydroxy-2,6-dioxohexanoate; DHDG, 2-dehydro-3-deoxy-d-gluconate; DHDG6P, 3-dehydro-3-deoxy-d-gluconate 6-phosphate; Ac-CoA, acetyl-CoA; uro, urocanate; Fo-Glu, formyl glutamate; CH_3_=H_4_F, methylene-tetrahydrofolate; CH≡H_4_F, methenyl-tetrahydrofolate; Fo-H_4_F, formyl-tetrahydrofolate; 2OB, 2-oxobutyrate; Prop-CoA, propionyl-CoA; ACAC, acetoacetate; GB-CoA, γ-amino-butyryl-CoA; But-CoA, butyryl-CoA; Fd, ferredoxin; XSH/X-S-S-X, thiol/disulfide pair; TCA, tricarboxylic acid cycle; PPP, pentose-phosphate pathway. **b**–**e**, NanoSIMS analysis of a highly purified MK-D1 culture incubated with a mixture of ^13^C- and ^15^N-labelled amino acids. **b**, Green fluorescent micrograph of SYBR Green I-stained cells. Aggregates are MK-D1, and filamentous cells are *Methanobacterium* sp. strain MO-MB1 (fluorescence can be weak owing to the high rigidity and low permeability of the cell membrane (Extended Data Fig. 2m, n; see also ref. ^49^). **c**, NanoSIMS ion image of ^12^C (cyan). **d**, NanoSIMS ion image of ^12^C^15^N/^12^C^14^N (magenta). **e**, Overlay image of **b**–**d**. **d**, The colour bar indicates the relative abundance of ^15^N expressed as ^15^N/^14^N. Scale bars 5 μm. The NanoSIMS analysis was performed without replicates due to its slow growth rate and low cell density. However, to ensure the reproducibility, we used two different types of highly purified cultures of MK-D1 (see Methods). Representative of *n* = 8 recorded images. The iTAG analysis of the imaged culture is shown in Supplementary Table 1.

**Extended Data Fig. 2:**
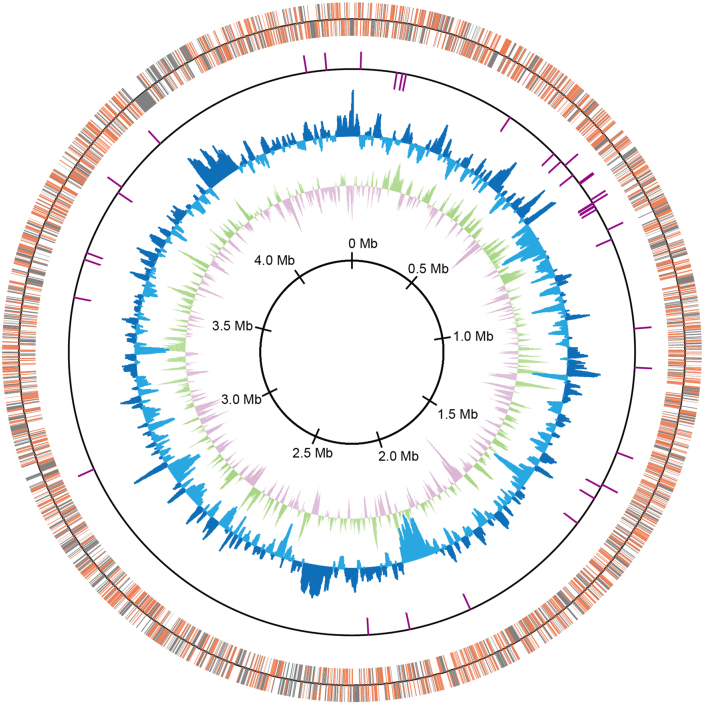
Circular representation of MK-D1 genome. From the outside to the centre: the distribution of the coding sequences based on the conserved (orange) or non-conserved (grey) genes in the first circle, non-coding RNAs in the second circle, GC content showing deviation from average (40.7%) in the third circle, and GC skew in the fourth circle. The GC content and GC skew were calculated using a sliding window of 2 kb in step of 10 kb. The coding sequences and RNA genes illustrate the findings for plus and minus strands.

**Extended Data Fig. 3:**
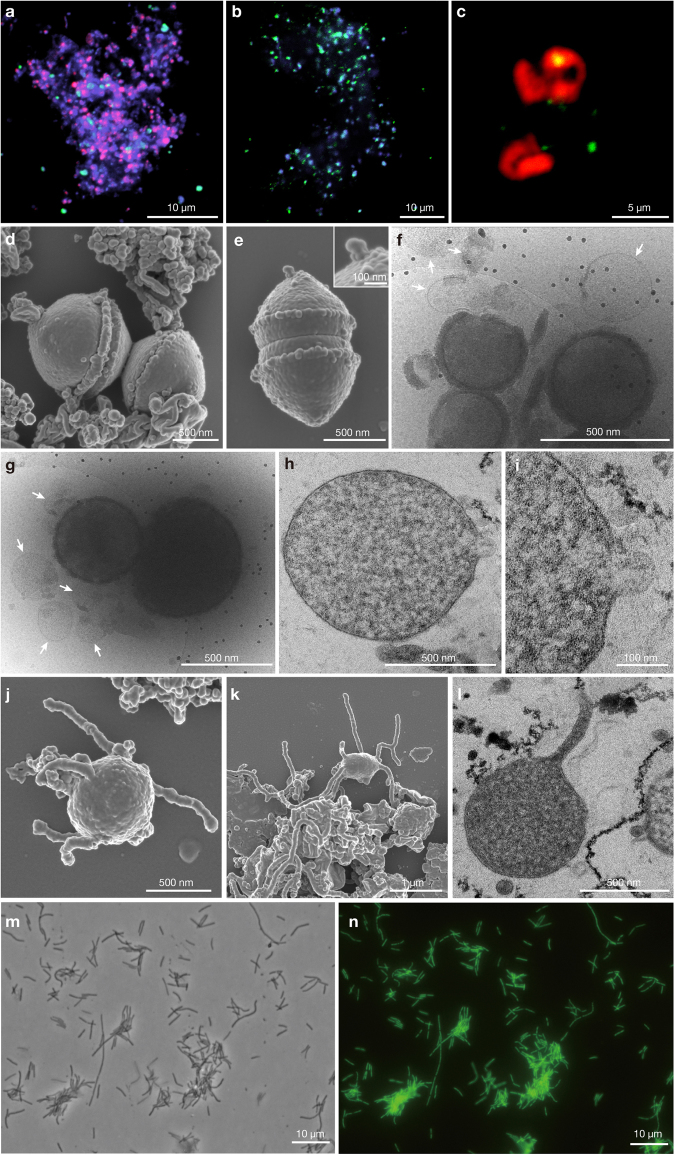
Other representative photomicrographs of MK-D1 cultures and *Methanobacterium* sp. strain MO-MB1. **a**, **b**, Fluorescence images of cells from enrichment cultures after 8 (**a**) and 11 (**b**) transfers stained with DAPI (violet) and hybridized with nucleotide probes that target MK-D1 (green) and Bacteria (red). The images are different fields of view to those shown in Fig. 1b, c, which were taken at the same time. **c**, A fluorescence image of cells in the enrichments after 11 transfers hybridized with nucleotide probes that target MK-D1 (green) and Archaea (but with one mismatch against MK-D1; red). Large and irregular coccoid-shaped cells stained by only ARC915 are probably *Methanogenium*. **d**, **e**, Dividing cells of MK-D1 with a bleb. The top-right inset image in **e** shows a magnification of the bleb. **f**, **g**, Cryo-EM images of MK-D1 cells and large membrane vesicles (white arrows). **h**, **i**, Ultrathin sections of MK-D1 cells with a membrane vesicle. The image **i** shows a magnified image of **h**. **j**, **k**, SEM images of MK-D1 cells with protrusions. **l**, Ultrathin section of a MK-D1 cell with a protrusion. **m**, **n**, Photomicrographs of pure culture of *Methanobacterium* sp. strain MO-MB1 cells stained with SYBR Green I. Phase-contrast (**m**) and fluorescence (**n**) images of the same field are shown. **a**, **b**, The FISH experiments were performed three times with similar results. **d**, **e**, **j**, **k**, The SEM images are representative of *n* = 122 recorded images that were obtained from four independent observations from four culture samples. The lipid composition experiments were repeated twice and gave similar results. **f**, **g**, The cryo-EM images are representative of *n* = 14 recorded images that were taken from two independent observations from two culture samples. **h**, **i**, **l**, The ultrathin-section images are representative of *n* = 131 recorded images that were obtained from six independent observations from six culture samples. **m**, **n**, The SYBR Green I staining experiment was performed once, but all 10 recorded images showed similar results. Detailed iTAG analyses of cultures are shown in Supplementary Table 1.

After six transfers, MK-D1 reached 13% abundance in a tri-culture containing a *Halodesulfovibrio* bacterium (85%) and a *Methanogenium* archaeon (2%) (Extended Data Table 1). Analyses using fluorescence in situ hybridization (FISH) and scanning electron microscopy (SEM) revealed a close physical association of the archaeon with the other microorganisms (Fig. 1b–e, Extended Data Fig. 3 and Supplementary Table 1). Through metagenome-based exploration of the metabolic potential of this archaeon and a stable-isotope probing experiment, we discovered that MK-D1 can catabolize ten amino acids and peptides through syntrophic growth with *Halodesulfovibrio* and *Methanogenium* through interspecies hydrogen (and/or formate) transfer^16^ (Fig. 2, Extended Data Fig. 2 and Supplementary Tables 2–4). Indeed, addition of hydrogen scavenger-inhibiting compounds (that is, 10 mM molybdate and 2-bromoethanesulfonate for sulfate-reducing bacteria (SRB) and methanogens, respectively) significantly impaired growth of MK-D1. Through subsequent transfers, we were able to eliminate the *Halodesulfovibrio* population, enabling us to obtain a pure co-culture of the target archaeon MK-D1 and *Methanogenium* after a 12-year study—from bioreactor-based pre-enrichment of deep-sea sediments to a final 7 years of in vitro enrichment. We here propose the name ‘*Candidatus* Prometheoarchaeum syntrophicum’ strain MK-D1 for the isolated archaeon (see Supplementary Note 3 for reasons why the provisional *Candidatus* status is necessary despite isolation).

## Cell biology, physiology and metabolism

**Fig. 3:**
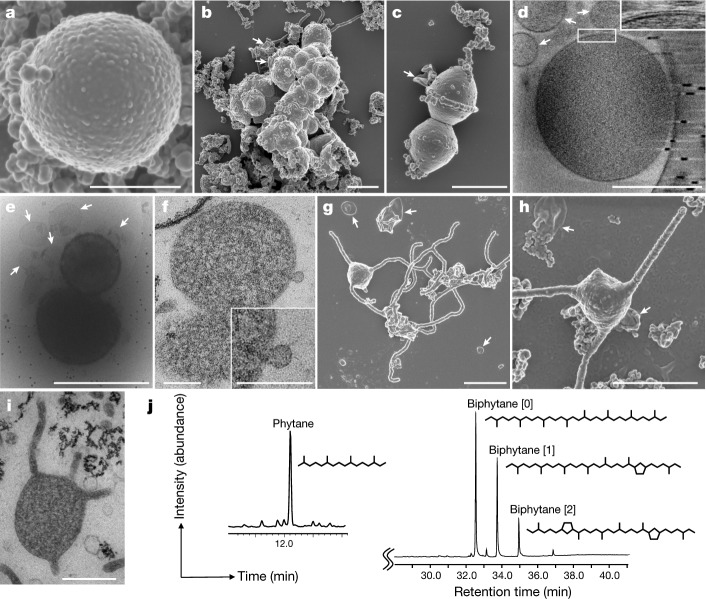
Microscopy characterization and lipid composition of MK-D1. **a**–**c**, SEM images of MK-D1. Single cell (**a**), aggregated cells covered with EPS-like materials (**b**) and a dividing cell with polar chains of blebs (**c**). **d**, Cryo-electron tomography image of MK-D1. The top-right inset image shows a magnification of the boxed area to show the cell envelope structure. **e**, Cryo-EM image of large membrane vesicles attached to and surrounding MK-D1 cells. **f**, Ultrathin section of an MK-D1 cell and a membrane vesicle. The bottom-right inset image shows a magnified view of the membrane vesicle. **g**, **h**, SEM images of MK-D1 cells producing long branching (**g**) and straight (**h**) membrane protrusions. **i**, Ultrathin section of a MK-D1 cell with protrusions. **j**, A total ion chromatogram of gas chromatography–mass spectrometry (GC–MS) for lipids extracted from a highly purified MK-D1 culture. The chemical structures of isoprenoids and their relative compositions are also shown (Supplementary Fig. 2). Scale bars, 1 μm (**b**, **c**, **g**, **h**), 500 nm (**a**, **d**, **e**, **i**) and 200 nm (**f**). **a**–**c**, **g**, **h**, SEM images are representative of *n* = 122 recorded images that were obtained from four independent observations from four culture samples. **d**, **e**, Cryo-EM images are representative of *n* = 14 recorded images that were taken from two independent observations from two culture samples. **f**, **i**, The ultrathin section images are representative of *n* = 131 recorded images that were obtained from six independent observations from six culture samples. White arrows in the images indicate large membrane vesicles. The lipid composition experiments were repeated twice and gave similar results. Detailed iTAG-based community compositions of the cultures are shown in Supplementary Table 1.

We further characterized MK-D1 using the pure co-cultures and highly purified cultures. Microscopy analyses showed that the cells were small cocci (approximately 300–750 nm in diameter (average, 550 nm)), and generally formed aggregates surrounded by extracellular polymer substances (EPS) (Fig. 3a, b and Extended Data Fig. 3), consistent with previous observations using FISH^15,17^. MK-D1 cells were easily identifiable given the morphological difference from their co-culture partner *Methanogenium* (highly irregular coccoid cells of ≥2 μm; Fig. 1d, e). Dividing cells had less EPS and a ring-like structure around the cells (Fig. 3c). Cryo-electron microscopy (cryo-EM) and transmission electron microscopy (TEM) analyses revealed that the cells contain no visible organelle-like inclusions (Fig. 3d–f and Supplementary Videos 1–6), in contrast to previous suggestions^6^. For cryo-EM, cells were differentiated from vesicles on the basis of the presence of cytosolic material (although DNA and ribosomes could not be differentiated), EPS on the cell surface and cell sizes that were consistent with observations by SEM and TEM analyses (Supplementary Videos 4–6). The cells produce membrane vesicles (50–280 nm in diameter) (Fig. 3b–f) and chains of blebs (Fig. 3c). MK-D1 cells also form membrane-based cytosol-connected protrusions of various lengths that have diameters of 80–100 nm, and display branching with a homogeneous appearance unlike those of other archaea (Fig. 3g–i; confirmed using both SEM and TEM). These protrusions neither form elaborate networks (as in *Pyrodictium*^18^) nor intercellular connections (*Pyrodictium*, *Thermococcus* and *Haloferax*^18–20^), suggesting differences in physiological functions. The MK-D1 cell envelope may be composed of a membrane and a surrounding S-layer, given the presence of four genes that encode putative S-layer proteins (Supplementary Fig. 1), stalk-like structures on the surface of the vesicles (Fig. 3e and Extended Data Fig. 3f, g) and the even distance between the inner and outer layers of the cell envelope (Fig. 3d). Lipid composition analysis of the MK-D1 and *Methanogenium* co-culture revealed typical archaeal isoprenoid signatures—C_20_-phytane and C_40_-biphytanes with 0–2 cyclopentane rings were obtained after ether-cleavage treatment (Fig. 3j). Considering the lipid data obtained from a reference *Methanogenium* isolate (99.3% 16S rRNA gene identity; Supplementary Fig. 2), MK-D1 probably contains C_20_-phytane and C_40_-biphytanes with 0–2 rings. The MK-D1 genome encoded most of the genes necessary to synthesize ether-type lipids—although geranylgeranylglyceryl phosphate synthase was missing—and lacked genes for ester-type lipid synthesis (Supplementary Tables 3, 4).

MK-D1 can degrade amino acids anaerobically, as confirmed by monitoring the depletion of amino acids during the growth of pure co-cultures (Extended Data Fig. 1b, c). We further verify the utilization of amino acids by quantifying the uptake of a mixture of ^13^C- and ^15^N-labelled amino acids through nanometre-scale secondary ion mass spectrometry (NanoSIMS) (Fig. 2b–e). Cell aggregates of MK-D1 incorporated amino-acid-derived nitrogen, demonstrating the capacity of MK-D1 to utilize amino acids for growth. Notably, the ^13^C-labelling of methane and CO_2_ varied depending on the methanogenic partner, indicating that MK-D1 produces both hydrogen and formate from amino acids for interspecies electron transfer (Extended Data Table 2). Indeed, addition of high concentrations of hydrogen or formate completely suppressed growth of MK-D1 (Extended Data Table 3). The syntrophic partner was replaceable—MK-D1 could also grow syntrophically with *Methanobacterium* sp. strain MO-MB1^21^ instead of *Methanogenium* (Fig. 2b–e). Although 14 different culture conditions were applied, none enhanced the cell yield, which indicates specialization of the degradation of amino acids and/or peptides (Extended Data Table 3).

To further characterize the physiology of the archaeon, we analysed the complete MK-D1 genome (Extended Data Fig. 2 and Supplementary Tables 2–6). The genome only encodes one hydrogenase (NiFe hydrogenase MvhADG–HdrABC) and formate dehydrogenase (molybdopterin-dependent FdhA), suggesting that these enzymes mediate reductive H_2_ and formate generation, respectively. MK-D1 represents, to our knowledge, the first cultured archaeon that can produce and syntrophically transfer H_2_ and formate using the above enzymes. We also found genes encoding proteins for the degradation of ten amino acids. Most of the identified amino-acid-catabolizing pathways only recover energy through the degradation of a 2-oxoacid intermediate (that is, pyruvate or 2-oxobutyrate; Fig. 2a and Supplementary Table 4). MK-D1 can degrade 2-oxoacids hydrolytically (through 2-oxoacid-formate lyases) or oxidatively (through 2-oxoacid:ferredoxin oxidoreductases) to yield acyl-CoA intermediates that can be further degraded for ATP generation. In the hydrolytic path, the carboxylate group of the amino acid is released as formate that can be directly handed off to partnering methanogenic archaea or SRB. In the oxidative path, 2-oxoacid oxidation is coupled with release of amino acid carboxylate as CO_2_ and reduction of ferredoxin, which can be re-oxidized through H^+^ and/or CO_2_ reduction to H_2_ and formate, respectively (through the electron-confurcating NiFe hydrogenase MvhADG–HdrABC or formate dehydrogenase FdhA). On the basis of ^13^C-amino-acid-based experiments (Supplementary Note 4), MK-D1 can probably switch between syntrophic interaction through 2-oxoacid hydrolysis and oxidation depending on the partner(s).

**Etymology**. *Prometheoarchaeum*, *Prometheus* (Greek): a Greek god who shaped humans out of mud and gave them the ability to create fire; *archaeum* from *archaea* (Greek): an ancient life. The genus name is an analogy between the evolutionary relationship this organism and the origin of eukaryotes, and the involvement of Prometheus in the origin of humans from sediments and the acquisition of an unprecedented oxygen-driven energy-harnessing ability. The species name, *syntrophicum*, *syn* (Greek): together with; *trephein* (Greek) nourish; *icus* (Latin) pertaining to. The species name refers to the syntrophic substrate utilization property of this strain.

**Locality**. Isolated from deep-sea methane-seep sediment of the Nankai Trough at 2,533 m water depth, off the Kumano area, Japan.

**Diagnosis**. Anaerobic, amino-acid-oxidizing archaeon, small coccus, around 550 nm in diameter, syntrophically grows with hydrogen- and formate-using microorganisms. It produces membrane vesicles, chains of blebs and membrane-based protrusions.

## Extant and ancestral features

**Fig. 4:**
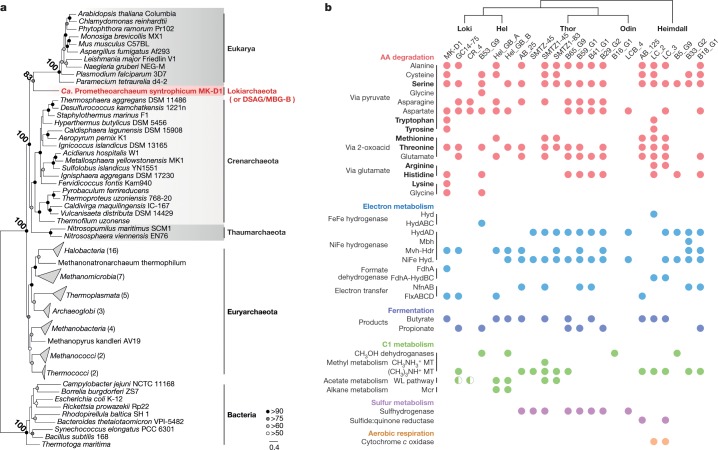
Phylogeny of MK-D1 and catabolic features of Asgard archaea. **a**, Maximum-likelihood tree (100 bootstrap replicates) of MK-D1 and select cultured archaea, eukaryotes and bacteria based on 31 ribosomal proteins that are conserved across the three domains (Supplementary Tables 7, 8). Bootstrap values around critical branching points are also shown. We used 14,024 sites of the alignment for tree construction. **b**, The presence or absence of amino acid degradation, electron metabolism, fermentation, C1 metabolism, sulfur metabolism and aerobic respiration in individual genomes are shown (complete pathway, full circle; mostly complete pathway, half circle). For amino acid metabolism, pathways that are exclusively used for catabolism or degradation are in bold. Glycine metabolism through pyruvate (top) or formate (bottom). Butyrate metabolism is reversible (fermentation or β-oxidation); however, butyryl-CoA dehydrogenases tend to be associated with EtfAB in the genomes, suggesting formation of an electron-confurcating complex for butyrate fermentation. Propionate was determined by the presence of methylmalonyl-CoA decarboxylase, biotin carboxyl carrier protein and pyruvate carboxylase. Propionate metabolism is also reversible; however, no member of the Asgard archaea encodes the full gene set for syntrophic degradation. Alcohol dehydrogenases can have diverse substrate specificities. See Supplementary Note 5 for abbreviations.

**Extended Data Fig. 4:**
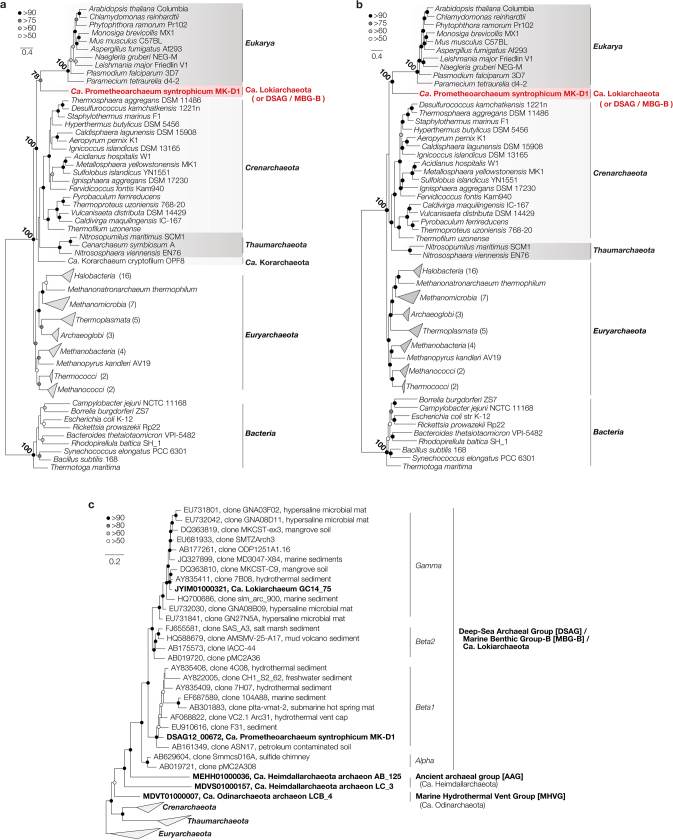
Ribosomal protein- and 16S rRNA gene-based phylogeny of MK-D1. **a**, Phylogenomic tree of MK-D1 and select cultured archaea, eukaryotes and bacteria based on 31 ribosomal proteins conserved across the three domains (Supplementary Table 7). Ribosomal protein sequences of MK-D1, the organisms shown in the tree and MAGs of uncultured archaeal lineages (Supplementary Table 8) were aligned individually using MAFFT. MAG-derived sequences (except for *Ca*. Korarchaeum) were then removed for tree construction. After removing all-gap positions and concatenation, the maximum-likelihood tree was constructed using RAxML-NG. Bootstrap values around critical branching points are also shown. In total, 14,875 sites of the alignment were used for tree construction. **b**, A ribosomal protein-based phylogenomic tree constructed using MrBayes. Bayesian inference phylogenies were calculated using MrBayes 3.2.7a and a ribosomal protein concatenated alignment used for Fig. 4a. **c**, Phylogenetic tree of MK-D1 and related archaea based on 16S rRNA genes. The 16S rRNA gene sequences were aligned using SINA against the Silva v.132 alignment and the maximum-likelihood tree was calculated using RAxML.

The evolutionary relationship between archaea and eukaryotes has been under debate, hinging on the incompleteness and contamination associated with metagenome-derived genomes and variation in results that depend on tree-construction protocols^22–25^. By isolating MK-D1, we were able to obtain a closed genome (Extended Data Fig. 2 and Supplementary Table 2) and construct ribosomal protein-based phylogenomic trees that show clear a phylogenetic sister relation between MK-D1 and Eukarya (Fig. 4a, Extended Data Fig. 4 and Supplementary Tables 7, 8). Thus, MK-D1 represents the closest cultured archaeal relative of eukaryotes. We confirmed the presence of 80 eukaryotic signature proteins, which are also observed in related Asgard archaea (Supplementary Figs. 3–13 and Supplementary Tables 3, 9). Moreover, RNA-based evidence for expression of such genes was obtained. Among eukaryotic signature proteins, 23 fall in the 500 most highly expressed genes, including hypothetical proteins related to actin, gelsolin, ubiquitin, ESCRT-III proteins (Vps2/24/46-like and Vps20/32/60-like), Roadblock/LC7 domain proteins and small GTP-binding domain proteins (Supplementary Tables 3, 9). Notably, MK-D1 simultaneously expresses three systems that could potentially contribute to cell division (FtsZ, actin and ESCRT-II/III; Supplementary Table 3).

**Extended Data Fig. 5:**
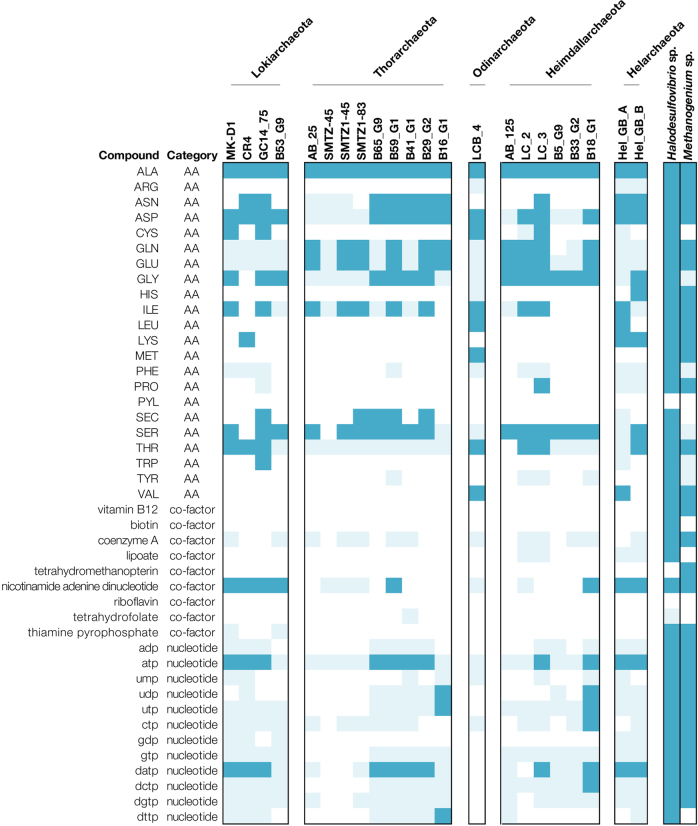
Amino acid, cofactor and nucleotide biosynthesis capacities of MK-D1 and other Asgard archaea. Genomes that encode proteins for the synthesis of amino acids, cofactors and nucleotides from pyruvate or acetyl-CoA (dark blue) and synthesis from other intermediates (light blue) are indicated. Those without complete pathways from pyruvate and/or acetyl-CoA are indicated in white. *Halodesulfovibrio* sp. strain MK-HDV and *Methanogenium* sp. strain MK-MG isolated in this study are also shown.

**Extended Data Fig. 6:**
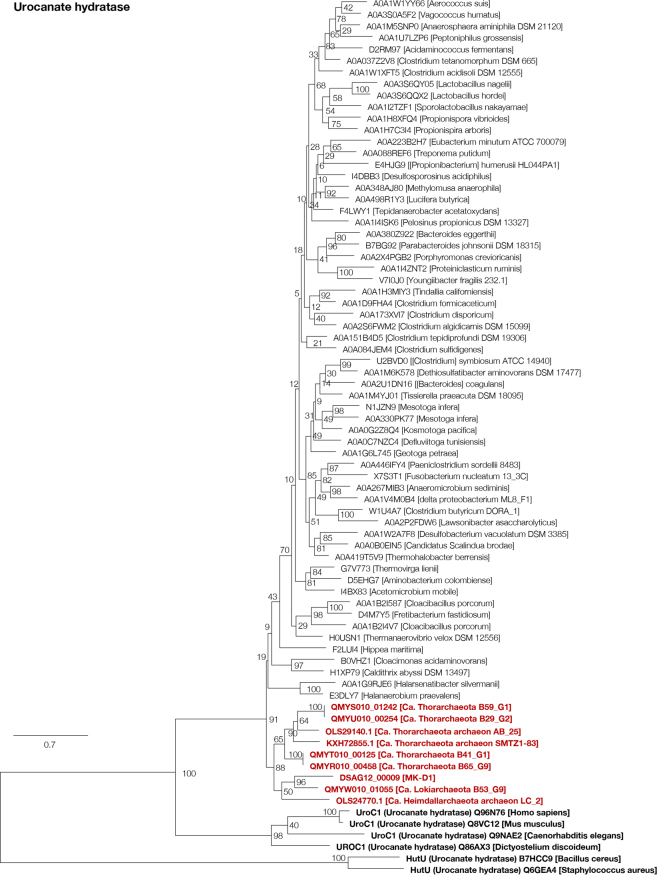
Maximum-likelihood tree of Asgard archaea urocanate hydratase. Urocanate hydratase (HutU) homologues were obtained by BLASTp analysis of the Asgard archaea sequences against the UniProt database (release 2019_06). Of homologues with sequence similarity ≥40% and overlap ≥70%, representative sequences were selected using CD-HIT with a clustering cut-off of 70% similarity (otherwise default settings were used). Additional homologues with verified biochemical activity, sequence similarity ≥30% and overlap ≥70% were obtained by BLASTp analysis of the Asgard archaea sequences against the UniProt/SwissProt database (2019_05). Sequences were aligned using MAFFT v.7 with default settings and trimmed using trimAl v.1.2 with default settings. The maximum-likelihood tree was constructed using RAxML-NG using fixed empirical substitution matrix (LG), 4 discrete GAMMA categories, empirical amino acid frequencies from the alignment and 100 bootstrap replicates. In total, 876 sites of the alignment were used for tree construction.

**Extended Data Fig. 7:**
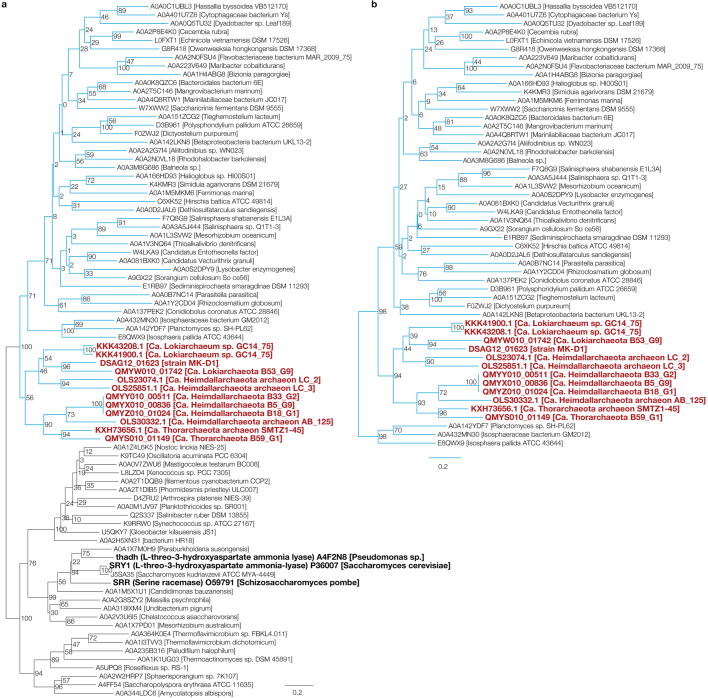
Maximum-likelihood tree of Asgard archaea l-threonine/l-serine dehydratase. **a**, Tree calculated for target Asgard archaea l-threonine/l-serine dehydratase (TdcB) and homologues. TdcB homologues were obtained by BLASTp analysis of the Asgard archaea sequences against the UniProt reference proteome and SwissProt database (release 2019_06). Of homologues with sequence similarity ≥40%, overlap ≥70% and predicted prosite domain PS00165 (serine/threonine dehydratases pyridoxal-phosphate attachment site), representative sequences were selected using CD-HIT with a clustering cut-off of 70% similarity (otherwise default settings were used). Additional homologues with verified biochemical activity, sequence similarity ≥30% and overlap ≥70% were obtained by BLASTp analysis of the Asgard archaea sequences against the UniProt/SwissProt database (2019_05). Sequences were aligned using MAFFT v.7 with default settings. Positions with gaps in more than 10% of the sequences were excluded from the alignment using trimAl v.1.2 (-gt 0.9; and otherwise default settings were used). The maximum-likelihood tree was constructed using PhyML using a fixed empirical substitution matrix (LG), 4 discrete GAMMA categories, empirical amino acid frequencies from the alignment and 100 bootstrap replicates (-b 100 -d aa -m LG -v e). In total, 308 sites of the alignment were used for tree construction. **b**, Tree calculated for a subset of sequences contained in a section of the original tree (branches that are coloured blue). Sequences were realigned and trimmed as described for **a**. In total, 308 sites of the alignment were used for tree construction.

Given the phylogenetic relationship of MK-D1, other Asgard archaea and eukaryotes, estimating the physiological traits of the last Asgard archaea common ancestor is of utmost importance. Comparative genomics between MK-D1 and published metagenome-assembled genomes of Asgard archaea revealed that most of the members encode amino-acid-catabolizing pathways, NiFe hydrogenases (MvhADG–HdrABC^26^ and/or HydAD^27^) (Fig. 4b), and have restricted biosynthetic capacities (that is, amino acid and vitamin synthesis; Extended Data Fig. 5), indicating that H_2_-evolving amino acid degradation and partner dependence may be a common feature across the superphylum. Like MK-D1, other members of the Asgard archaea possess enzymes associated with syntrophic bacteria (the electron transfer complex FlxABCD–HdrABC^28^ and formate dehydrogenases), indicating that other archaea have the capacity to degrade amino acids syntrophically. Many lineages also possess genes for fermentative propionate and/or butyrate production (Fig. 4b). Various other unique types of metabolism can be identified (for example, mono/tri-methylamine-driven homoacetogenesis and coupled H_2_/S^0^ metabolism in Thorarchaeota; H_2_S metabolism in Heimdallarchaeota; other types have been reported by other studies^6–8,10,11,14^), but are either only sporadically present or confined to specific phylum-level lineages. To identify potential ancestral features, we searched for catabolic genes that are conserved across phylum-level lineages including Heimdallarchaeota (currently the most deep-branching Asgard archaea) that form monophyletic clusters. We found key catabolic genes for histidine, serine and threonine degradation (urocanate hydratase and serine/threonine dehydratase; Extended Data Figs. 6, 7), butyrate fermentation (fatty-acid-CoA ligase and 3-ketoacyl-CoA thiolase; Supplementary Figs. 14, 15) and propionate fermentation (succinate dehydrogenase flavoprotein subunit, methylmalonyl-CoA transcarboxylase-associated biotin ligase and biotin carboxyl carrier protein; Supplementary Figs. 16–18). Given the physiology of the isolated MK-D1; the presence of amino acid catabolism and H_2_ metabolism and the lack of biosynthetic pathways in nearly all extant Asgard archaea lineages; and conservation of the above metabolism types, we propose that the last Asgard archaea common ancestor was an amino-acid-degrading anaerobe that produced H_2_ and fatty acids as by-products, acquired ATP primarily from substrate-level phosphorylation through catabolizing 2-oxoacid intermediates and depended on metabolic partners, although we do not reject the possibility of other additional lifestyles. In summary, we provide evidence that Asgard archaea are capable of syntrophic degradation of amino acids, are dependent on symbiotic interactions for both catabolism and anabolism (for example, H_2_, formate and metabolite transfer) and conserve related fermentative metabolic features across the superphylum, suggesting that the ancestor of the Asgard archaea possessed such capacities. This shows some congruence with a previous study that proposes hydrogenogenesis as a feature of the ancestor^12^, but differs in several central features.

## New insights into eukaryogenesis

The origin of the eukaryotic cell is one of the most enigmatic questions in biology. Isolation and cultivation of MK-D1 brings us closer to understanding how eukaryotes may have emerged; however, it is important to emphasize that the vast amount of time (roughly 2 billion years) that separates this modern-day organism from the organism that evolved into the last eukaryotic common ancestor (LECA) leaves many uncertainties—although we can make reasoned assumptions on the events that may have occurred during the course of evolution. The discussion that follows is a hypothetical model, in which we build on existing hypotheses with extrapolations from the insights gained in this study; notably, the model is not definitive and more studies on Asgard archaea and other deep-branching eukaryotes are required to contextualize the most probable steps that occurred.

**Fig. 5:**
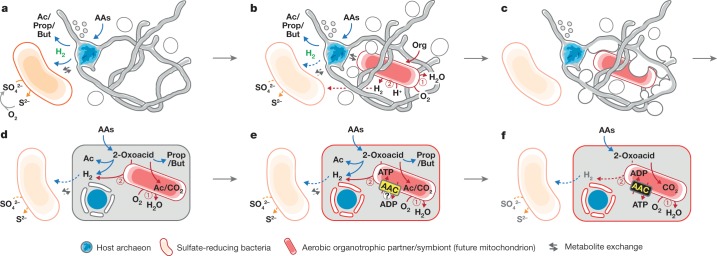
Proposed hypothetical model for eukaryogenesis. **a**, The syntrophic/fermentative host archaeon is suggested to degrade amino acids to short-chain fatty acids and H_2_, possibly by interacting with H_2_-scavenging (and indirectly O_2_-scavenging) SRB (orange; see Supplementary Note 6). **b**, The host may have further interacted with a facultatively aerobic organotrophic partner that could scavenge toxic O_2_ (the future mitochondrion; red). Continued interaction with SRB could have been beneficial but not necessarily essential; dotted arrows indicate the interaction; see Supplementary Note 7. **c**, Host external structures could have interacted (for example, mechanical or biological fusion^50^) with the aerobic partner to enhance physical interaction and further engulf the partner for simultaneous development of endosymbiosis and a primitive nucleoid-bounding membrane. **d**, After engulfment, the host and symbiont could have continued the interaction shown in **b** as a primitive type of endosymbiosis. **e**, Development of ADP/ATP carrier (AAC) by the endosymbiont (initial direction of ATP transport remains unclear; see Supplementary Note 9). **f**, Endogenization of partner symbiosis by the host through delegation of catabolism and ATP generation to the endosymbiont and establishment of a symbiont-to-host ATP channel.

Assuming that the ancestor of the Asgard archaea was indeed syntrophic, internally simple (that is, similar to MK-D1) and inhabited anaerobic marine sediments as most of the extant members of this lineage do^6^, evolution towards the facultatively aerobic LECA^29^ can be envisioned to require (1) transition from anaerobiosis to aerobiosis, (2) the gain of an O_2_-respiring and ATP-providing endosymbiont (that is, mitochondrion), and (3) development of intracellular structures. As Earth’s O_2_ levels^30^ had begun to rise before the evolution of the LECA (the TACK-Asgard archaea lineage dated to approximately 2.1–2.4 billion years ago^31^), we work on the assumption that the archaea needed to accommodate the increasing O_2_ levels, and energy and organic substrate inputs^32^, especially in benthic habitats of shallow oceans. Aerotolerance might have been conferred by a symbiotic interaction with facultative O_2_-respiring organisms^33,34^, which was potentially followed by endosymbiosis of one of these aerobes (that is, the future mitochondrion). Although such a transition from syntrophy to aerobiosis is non-trivial, we suggest that a syntrophic interaction with SRB could have mediated this (Fig. 5a, b and Supplementary Notes 6, 7). Although previous models propose that H_2_ transfer was a key interaction that drove endosymbiosis^12,29,35,36^, we believe that current data favours the above interaction (see Supplementary Note 8). Given the small cell size of MK-D1 and the proposed lack of sufficient machinery^37^ and energy^38^, we suggest that the physical manifestation of this endosymbiosis was probably independent of phagocytosis^6^. The observed morphology of strain MK-D1 rather points to a previously proposed alternative route^39^ in which the host archaeon engulfed the metabolic partner using extracellular structures and simultaneously formed a primitive chromosome-surrounding structure that is topologically similar to the nuclear membrane; however, further evidence is required to support this conjecture (Fig. 5c, d).

After engulfment, the host may have shared amino-acid-derived 2-oxoacids with the endosymbiont as energy sources (Fig. 5d), given that amino-acid-degrading pathways widely encoded by Asgard archaea primarily recover ATP from 2-oxoacid degradation (Fig. 4b) and extant eukaryotes and mitochondria share 2-oxoacids^40^. In return, the endosymbiont may have consumed O_2_ (as proposed previously^33^) and provided the host with an intracellular pool of biological building blocks (for example, amino acids and co-factors that the host may not have been able to synthesize that were released passively or through endosymbiont death). On the basis of the absence of host-derived (that is, archaea-related) anaerobic 2-oxoacid catabolism genes (for example, ferredoxin-dependent 2-oxoacid oxidoreductase and NiFe hydrogenases) in eukaryotes^41,42^, the host presumably lost these during evolution towards the LECA. Notably, this loss might have consequently helped to simultaneously resolve catabolic redundancy (that is, 2-oxoacid catabolism in both host and symbiont) and O_2_ sensitivity (that is, O_2_ inactivates these enzymes^43,44^). For the resulting delegation of 2-oxoacid catabolism (and thus ATP generation) to the endosymbiont (as in modern mitochondria) to succeed, an ATP transport mechanism would have been necessary. Consistent with this notion, evolution of the ATP transporter (that is, the ADP/ATP carrier^45^) is thought to have been instrumental in fixing the symbiosis^46^ (see Supplementary Note 9 for potential impetus; Fig. 5e). Another transition may have been necessary—the host archaeon may have possessed ether-type lipids as observed for MK-D1 (Fig. 3j) and Asgard archaea^47^, yet all extant eukaryotes use ester-type lipids. However, a recent study showed that lipid types can mix without losing membrane integrity^48^, suggesting that the simple replacement of host ether-type lipids with ester-type lipids may have been possible (Fig. 5e). This hypothetical evolutionary scenario may have provided the steps that are required for the emergence of an aerobic organotroph that possess an O_2_-respiring ATP-generating endosymbiont congruent with extant eukaryotes and their mitochondria in terms of energy metabolism (Fig. 5f).

In summary, we have isolated and cultivated the closest archaeal relative of eukaryotes to date that has a unique metabolism and morphology, and combining these observations with genomic analyses, propose the entangle–engulf–endogenize model as one of several conceivable scenarios to explain the emergence of eukaryotes. Further investigation of MK-D1, related Asgard archaea and more deep-branching eukaryotes is now required and can provide valuable insights into the timing and progression of lateral gene transfer, endosymbiont organellogenesis towards the first mitochondrion and the formation of the endomembrane system (among many other physiological features). Such endeavours are essential to refine our understanding of the possible chain of events that led to the eukaryotic cell, and to provide the necessary data that support or refute our models of eukaryogenesis.

## Methods

No statistical methods were used to predetermine sample size. The experiments were not randomized. The investigators were not blinded to allocation during experiments and outcome assessment.

### Sampling site and sample description

A 25-cm long sediment core (949C3) was collected from a methane-seep site at the Omine Ridge, Nankai Trough, off the Kumano area, Japan (33° 7.2253′ N, 136° 28.6672′ E), 2,533 m below sea level, by the manned submersible RV *Shinkai 6500* (cruise YK06-03, dive no. 6K949, 6 May 2006). The detailed sediment core sample and site information has been published previously^15,51,52^. Our previous geochemical and 16S rRNA gene analysis indicated that the occurrence of anaerobic oxidation of methane reactions was mediated by archaeal anaerobic methanotrophs in the sediment^15,51^. The SSU rRNA gene analysis also showed that the sediment contained abundant and diverse microorganisms, most of which were affiliated with uncultured microbial groups, including Asgard archaea^15,51^.

### Culturing

The deep-sea methane-seep sediment sample was first enriched using a continuous-flow bioreactor system supplemented with methane as the major energy source. The bioreactor, called a down-flow hanging sponge (DHS) bioreactor, has been operated in our laboratory, JAMSTEC, Yokosuka Headquarters, since 28 December 2006. The detailed operation conditions for the DHS bioreactor have been described previously^15^. To isolate anaerobic microorganisms, including Asgard archaea, from the DHS reactor, 2-ml samples of the bioreactor enrichment sediment slurry were inoculated in 15-ml glass tubes with a simple substrate and a basal medium. The composition of the basal medium was almost similar to that used for cultivation in the DHS bioreactor^15^, but it did not contain sulfate (that is, Na_2_SO_4_). The basal medium composition was as follows (per litre): 9.47 g MgCl_2_·6H_2_O, 1.36 g CaCl_2_·2H_2_O, 20.7 g NaCl, 0.54 g NH_4_Cl, 0.14 g KH_2_PO_4_, 2.7 g NaHCO_3_, 0.3 g Na_2_S·9H_2_O, 0.3 g cysteine·HCl, 1 ml trace element solution^15^, 1 ml Se/W solution, 2 ml vitamin solution^15^ and resazurin solution (1 mg ml^−1^). The medium was purged with N_2_/CO_2_ gas (80:20, v/v), and the pH was adjusted to 7.5 at 25 °C. The culture tubes were sealed with butyl rubber stoppers and screw caps. Autoclaved or filter-sterilized organic substances (such as protein-derived materials, sugars and fatty acids) were added to the tubes with stock solutions before inoculation with the bioreactor-enriched community. After establishing a stable *Ca*. P. syntrophicum culture, cultivations were performed at 20 °C in 50-ml serum vials containing 20 ml basal medium supplemented with casamino acids (0.05%, w/v), 20 amino acids (0.1 mM each) and powdered milk (0.1%, w/v, Hohoemi, Meiji) under an atmosphere of N_2_/CO_2_ (80:20, v/v) in the dark without shaking, unless mentioned otherwise. Information regarding the purity check of MK-D1 cultures, as well as additional information about cultivation, is included in the Supplementary Methods.

### SSU rRNA gene-based analysis

DNA extraction and PCR mixture preparation were performed on a clean bench to reduce contamination. DNA extraction from culture samples was performed as described previously^53^. The concentration of extracted DNA was measured using a Quant-iT dsDNA High-Sensitivity Assay Kit (Life Technologies). PCR amplification was performed using the Takara Ex *Taq* (for conventional clone analysis) or Takara LA *Taq* (for Illumina-based amplicon sequencing (iTAG) for targeted sequencing for the SSU rRNA gene analysis) (Takara Bio), and the reaction mixtures for PCR were prepared according to the manufacturer’s instructions. For the conventional clone analysis, a universal primer pair 530F/907R^51^ and an archaeal primer pair 340F/932R^15,54^ were used for PCR amplification. For iTAG analysis, the universal primer pair 530F/907R, which contained overhang adapters at the 5′ ends, was used. The procedures used for library construction, sequencing and data analysis were described previously^21,55^.

### Growth monitoring using qPCR

For the quantitative analysis, a StepOnePlus Real-Time PCR System (Thermo Fisher Scientific) with a SYBR Premix Ex Taq II kit (TaKaRa Bio) was used. The candidate phylum Lokiarchaeota-specific primer pair MBGB525F/Ar912r was used for amplification of 16S rRNA genes. Primer MBGB525F is the complementary sequence of the MGBG525 probe^17^, whereas Ar912r is an archaeal universal primer that is a slightly modified version of the originally designed primer^56^. The detailed procedure for qPCR is described in the Supplementary Methods. The doubling times of MK-D1 were calculated based on the semi-logarithmic plot of the qPCR data.

### Growth test with multiple substrates

To examine the effect of the presence of other substances on the growth of MK-D1, medium containing casamino acids, 20 amino acids, powdered milk and supplemented with an individual substrate (Extended Data Table 3) was prepared, followed by qPCR and iTAG analyses. Each cultivation condition was set in duplicate; however, the H_2_-fed culture was prepared in triplicate because a previous study^7^ reported that a Lokiarchaeum has potential to grow with hydrogen based on a comparative genome analysis. Detailed culture liquid sampling and the subsequent qPCR and iTAG analyses are described in the Supplementary Information.

### Evaluation of growth temperature

The test was performed using a basal medium containing casamino acids and powdered milk, with a pure co-culture of MK-D1 and *Methanogenium* as the inoculum (20%, v/v). The cultures were incubated at 4, 10, 15, 20, 25, 30, 37 and 40 °C. All incubations for the test were performed in triplicate. After 100 days of incubation, 16S rRNA gene copy numbers of MK-D1 were evaluated using qPCR.

### FISH

Fixation of microbial cells, storage of the fixed cells and standard FISH were performed in accordance with a previously described protocol^21^. The 16S rRNA-targeted oligonucleotide probes used in this study are listed in Supplementary Table 10. The design of MK-D1-specific probes is described in the Supplementary Methods. As clear fluorescent signals were not obtained using the standard FISH technique, we used an in situ DNA-hybridization chain reaction (HCR) technique^57^. The FISH samples were observed using epifluorescence microscopes (BX51 or BX53, Olympus) and a confocal laser scanning microscope (Nikon A1RMP, Nikon Instech).

### SEM

Microbial cells were fixed overnight in 2.5% (w/v) glutaraldehyde in the casamino acids–20 amino acid medium at 20 °C. The sample preparation procedure has been described previously^58^. The cell samples were observed using field emission-SEM (JSM-6700F, JEOL) or extreme high-resolution FIB-SEM (Helios G4 UX, ThermoFisher Scientific).

### Ultrathin sectioning and TEM

Cells were prefixed with 2.5% (w/v) glutaraldehyde for 2 h. The specimens were frozen in a high-pressure freezing apparatus (EM-PACT2, Leica)^59^. The frozen samples were substituted with 2% OsO_4_ in acetone for 3–4 days at −80 °C, and the samples were warmed gradually to room temperature, rinsed with acetone embedded in epoxy resin (TAAB). Thin sections (70 nm) were cut with am ultramicrotome (EM-UC7, Leica). Ultrathin sections of the cells were stained with 2% uranyl acetate and lead-stained solution (0.3% lead nitrate and 0.3% lead acetate, Sigma-Aldrich), and were observed using TEM (Tecnai 20, FEI) at an acceleration voltage of 120 kV.

### Cryo-EM

Owing to the low cell yield culture, 400 ml of the culture of MK-D1 was prepared and concentrated to about 5 ml using a 0.22-μm-pore-size polyethersulfone filter unit (Corning) in an anaerobic chamber (95:5 (v/v) N_2_:H_2_ atmosphere; COY Laboratory Products). The concentrated culture liquid was placed in a glass vial in the anaerobic chamber. After that, the head space of the glass vial was replaced by N_2_/CO_2_ gas (80:20, v/v). Immediately before the observation using electron microscopy, the glass vial was opened, and the liquid culture was concentrated to about 200 μl by centrifugation at 20,400*g* for 10 min at 20 °C. Subsequently, 3 μl of the concentrated liquid culture was applied onto a Quantifoil Mo grid R1.2/1.3 (Quantifoil MicroTools) pretreated with glow-discharge, and was plunged-frozen in liquid ethane using a Vitrobot Mark IV (FEI Company) at 4 °C and 95% humidity.

The frozen grid was mounted onto a 914 liquid-nitrogen cryo-specimen holder (Gatan) and loaded into a JEM2200FS electron microscope (JEOL) equipped with a field emission electron source operating at 200 kV and an omega-type in-column energy filter (slit width: 20 eV). The images were recorded on a DE-20 direct detector camera (Direct Electron) at a nominal magnification of 15,000×, which resulted in an imaging resolution of 3.66 Å per pixel, with the total dose under 20 electrons per Å^2^ using a low-dose system. For electron tomography, tilt series images were collected manually in a range of approximately ±62° at 2° increments. The total electron dose on the specimen per tilt series was kept under 100 electrons per Å^2^ to minimize radiation damage. The tilt series were aligned using gold fiducials and tomograms were reconstructed using filtered back projection or SIRT in the IMOD software^60^ with an image binning of 5.

### Lipid analysis

About 120 ml of a highly purified culture sample was concentrated using the same method as described above, except that the filtration concentration procedure was performed on a clean bench instead of the anaerobic chamber. After cell collection, the cells were washed with the anaerobic basal medium to eliminate the interfering matrix. Subsequently, lipid analysis was conducted for the collected cells after the improved method^61^. For precise qualitative liquid analysis, GC–MS was conducted on the 7890 system (Agilent Technologies) to compare the retention time and mass fragmentation signatures.

### Stable isotope probing and NanoSIMS analysis

To confirm utilization of amino acids by MK-D1, a stable-isotope probing experiment was performed using a ^13^C- and ^15^N-labelled amino acid mixture (Cambridge Isotope Laboratories). In brief, 120 ml serum vials containing 40 ml basal medium were prepared and supplemented with the 20 stable-isotope-labelled amino acids (roughly 0.1 mM of each), casamino acids (0.05%, w/v) and non-labelled 20 amino acid mixture (0.1 mM of each). Two types of highly purified cultures of MK-D1 were used as inocula: a co-culture with *Methanobacterium* sp. strain MO-MB1 and a tri-culture with *Halodesulfovibrio* and *Methanogenium*. The vials were incubated at 20 °C in the dark without shaking for 120 days. A reference cultivation was also performed under the same cultivation conditions without the addition of the 20 stable-isotope-labelled amino acid mixture (Extended Data Table 2). The detailed sample preparation and analysis method using NanoSIMS is described in the Supplementary Methods.

### Chemical analysis

The stable carbon isotope compositions of methane and CO_2_ in the sampled gas phase were analysed as described previously^62^. Methane concentrations were measured by GC (GC-4000, GL Science) using a Shincarbon ST 50/80 column (1.0 m × 3.0 mm inner diameter; Shinwa Chemical Industries) and a flame ionization detector with nitrogen as a carrier gas.

Amino acid concentrations in pure co-cultures of MK-D1 and *Methanogenium* were quantified through a previously described method^63,64^. In brief, we processed the acid hydrolysis with 6 M HCl (110 °C, 12 h) for the culture liquid samples after filtration using a 0.2-μm pore-size polytetrafluoroethylene filter unit (Millipore). The amino acid fraction was derivatized to *N*-pivaloyl iso-propyl esters before GC using a 6890N GC instrument connected to the nitrogen phosphorus and flame ionization detectors (Agilent Technologies). For cross-validation of qualitative identification of amino acids, GC–MS on the 7890 system (Agilent Technologies) was used^61^.

### Genome sequencing and assembly

DNA extraction was performed as described previously^53^. Mate-paired library with an average insert size of 3,000 bp was constructed according to the manufacturer’s instructions with Nextera Mate Pair Library Preparation kit (Illumina). Library sequencing was performed using Illumina MiSeq platform (2 × 300 bp), which resulted in 3,822,290 paired reads. The mate pair reads were processed as follows: adapters and low-quality sequences were removed using Trimmomatic v.0.33^63^ (ILLUMINACLIP:TruSeq3-PE-2.fa:2:30:10:8:true LEADING:3 TRAILING:3 SLIDINGWINDOW:4:20 MINLEN:100), and the linker sequences were removed using NextClip v.1.3.1^65^. De novo assembly was performed using SPAdes v.3.1.1^66^ with multiple *k*-mer sizes (21, 33, 55, 77 and 99), which resulted in 3,487 contigs with lengths >500 bp, totalling up to 14.68 Mb. The software MyCC^67^ was used with default parameters for binning based on genomic signatures, marker genes and contig coverages. As heterogeneity in the sequence can cause highly fragmented or redundant contigs, the ambiguous contigs (sequence coverage <5 or a length < 1kb) and redundant contigs were discarded from binning. This resulted in the recovery of genomes related to Lokiarchaeota (that is, *Ca*. P. syntrophicum MK-D1, 4.46 Mb), *Halodesulfovibrio* (4.13 Mb) and *Methanogenium* (2.33 Mb). Scaffolds for each bin were constructed using SSPACE v.3.0^68^ with mate-paired information of Illumina reads. To obtain the complete genome sequence of *Ca*. P. syntrophicum, the gaps were filled using Sanger sequencing. Genomes were annotated using Prokka v.1.12^69^ and manually curated. The curation involved functional domain analysis through CD-Search (CDD v.3.17) with its corresponding conserved domain database^70,71^ and InterProScan v.5^72^; signal peptide and transmembrane domain prediction through SignalP v.4.1^73^; carbohydrate-active enzyme, peptidase and lipase prediction through dbCAN v.5.0^74^, MEROPS^75^ and lipase engineering database^76^; and hydrogenase annotation with assistance from HydDB^77^. In addition, to further verify the function, we compared the sequence similarity of each gene to enzymes found in UniProtKB/SwissProt that had experimentally verified catalytic activity and genes with extensive genetic, phylogenetic and/or genomic characterizations^78,79^ with a 40% amino acid similarity cut-off. For enzymes that have divergent functions even with a 40% similarity cut-off (for example, [FeFe] and [NiFe] hydrogenases, 3-oxoacid oxidoreductases, glutamate dehydrogenases and sugar kinases), phylogenetic trees were constructed with reference sequences to identify association of the query sequences to phylogenetic clusters containing enzymes with characterized catalytic activity. Publicly available metagenome-assembled genomes of Asgard archaea were annotated in the same manner.

### Phylogenetic analysis

Phylogenomic trees of MK-D1 and select cultured archaea, eukaryotes and bacteria were calculated. Thirty-one ribosomal proteins conserved across the three domains (Supplementary Table 7) were collected from MK-D1, the organisms shown in the tree and metagenome-assembled genomes (MAGs) of uncultured archaeal lineages (Supplementary Table 8). Two alignments were performed in parallel: (1) only including sequences from cultured organisms and (2) also including MAG-derived sequences. MAFFT v.7 (--linsi) was used for alignment in both cases^80^. For the latter, MAG-derived sequences were included to generate an alignment that maximizes the archaeal diversity that is taken into account, but removed for subsequent tree construction to avoid any influence of contamination (that is, concatenation of sequences that do not belong to the same organism). ‘*Candidatus* Korarchaeum’ sequences were kept in the tree based on the cultured + uncultured alignment due to its critical position in TACK phylogeny. After removing all-gap positions and concatenation, the maximum-likelihood trees were constructed using RAxML-NG v.0.8.0^81^ (fixed empirical substitution matrix (LG), 4 discrete GAMMA categories, empirical amino acid frequencies and 100 bootstrap replicates) and the Bayesian inference phylogenies were calculated using MrBayes v.3.2.7a^82^ (four chains, print/sample frequencies of 100, a relative burn-in of 25% (nchains = 4 nruns = 2 printfreq = 100 samplefreq = 100), LG model, invariable sites plus GAMMA models of rate variation across sites (prset aamodellpr = fixed(lg); lset rates = invgamma)). For 16S ribosomal RNA phylogeny, sequences were aligned using SINA^83^ against the Silva v.132 alignment^84^. The maximum-likelihood tree was calculated using RAxML^85^ using the same parameters as RAxML-NG.

For analysis of urocanate hydratase, serine/threonine dehydratase, succinate dehydrogenase flavoprotein, fatty-acid-CoA ligase and 3-ketoacyl-CoA thiolase, homologues were collected through BLASTp^86^ analysis of the Asgard archaea sequences against the UniProt database (release 2019_05). Asgard archaea protein sequences unavailable in GenBank or UniProt (that is, those without accession numbers in the trees) were predicted with Prokka v.1.13^69^ (--kingdom Archaea --rnammer) using the genome assemblies available in GenBank. Of homologues with sequence similarity ≥40% and overlap ≥70%, representative sequences were selected using CD-HIT v.4.8.1^87^ with a clustering cut-off of 70% similarity (default settings otherwise). Additional homologues with verified biochemical activity, sequence similarity ≥30%, and overlap ≥70% were collected through BLASTp^86^ analysis of the Asgard archaea sequences against the UniProt/SwissProt database (2019_05)^88^. Sequences were aligned using MAFFT v.7^80^ with default settings (or MUSCLE v.3.8.31^89^ where noted) and trimmed using trimAl v.1.2^90^ (settings are specified in the caption for each corresponding phylogenetic tree). RAxML-NG^81^ was used for tree construction with the same parameters above (or PhyML v.3.3^91^ with 100 bootstrap replicates, LG model and empirical amino acid frequencies where noted). For analysis of biotin ligase and biotin carboxyl carrier protein, the phylogenetic tree was constructed using FastTree^92^ using the LG model and 1,000 bootstrap replicates.

### RNA-based sequencing analysis

To perform RNA-based sequencing analysis, 100 ml of culture liquid was prepared from 5 highly purified cultures that were incubated with casamino acids, 20 amino acids and powdered milk for about 100 days at 20 °C. Before RNA extraction, the growth of MK-D1 was confirmed using qPCR, and the cells density levels were around 10^5^ copies ml^−1^ in each culture.

To collect microbial cells, the culture liquid was filtered through a 0.22-μm pore-size mixed cellulose ester membrane filter (GSWP01300, Merck MilliPore) on a clean bench. After filtration, the membrane was cut in half with sterilized scissors and then directly inserted into the PowerBiofilm bead tubes of a PowerBiofilm RNA Isolation kit (MO BIO Laboratories). The following RNA extraction procedures were performed according to the manufacturer’s instructions. The extracted RNA was applied to an RNA Clean & Concentrator Kit-5 (Zymo Research) for concentration. The obtained RNA was quantified using an Agilent 2100 Bioanalyzer system with an RNA Pico kit (Agilent Technologies) and then applied to an Ovation Universal RNA-Seq System (NuGEN Technologies) for the construction of an RNA-sequence library. At the step for Insert Dependent Adaptor Cleavage technology-mediated adaptor cleavage during the library construction, specific primers for 16S rRNA and 23S rRNA genes of MK-D1 were used to reduce rRNA gene sequences from the cDNA pool. The constructed cDNA library was sequenced using the MiSeq platform (Illumina).

The raw RNA sequencing data were trimmed by removal of the adapters and low-quality sequences using Trimmomatic v.0.33^93^. The expression abundance of all coding transcripts was estimated in RPKM values using EDGE-pro v.1.3.1^94^.

### Reporting summary

Further information on research design is available in the Nature Research Reporting Summary linked to this paper.

## Online content

Any methods, additional references, Nature Research reporting summaries, source data, extended data, supplementary information, acknowledgements, peer review information; details of author contributions and competing interests; and statements of data and code availability are available at 10.1038/s41586-019-1916-6.

### Supplementary information

Supplementary Information This file contains Supplementary Notes 1–9, Supplementary Methods, Supplementary Figures 1–18, and Supplementary References.

Reporting Summary

Supplementary Tables This file contains Supplementary Tables 1–10

Supplementary Video 1 | Tilt-series images of a single cell of MK-D1

Supplementary Video 2 | Z-slices of the tomographic three-dimensional reconstruction from the tilt-series in Supplementary Video 1

Supplementary Video 3 | Animation of the same MK-D1 cell as in Supplementary Video 2 The cell envelope and membrane vesicles are colored in light blue and pink, respectively.

Supplementary Video 4 | Tilt-series images of MK-D1 cells

Supplementary Video 5 | Z-slices of the tomographic three-dimensional reconstruction from the tilt-series in Supplementary Video 4

Supplementary Video 6 | Animation of the same MK-D1 cells as in Supplementary Video 5 The cell envelope and membrane vesicles are colored in light blue and pink, respectively.

## Figures and Tables

**Extended Data Table 1:**
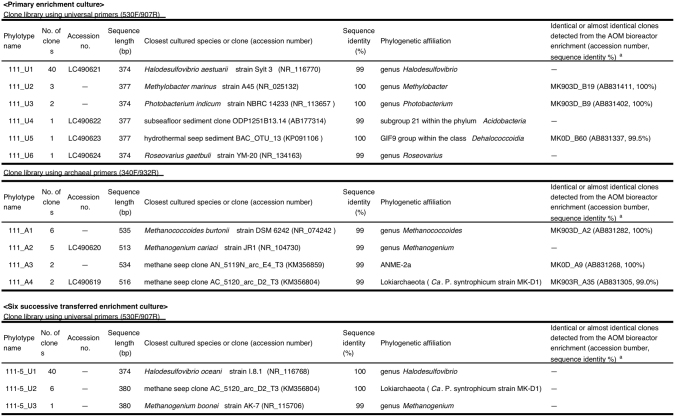
SSU rRNA gene clones obtained from the primary and six successive transferred enrichment cultures

^a^The clone sequences have been reported in our previous study^15^.

**Extended Data Table 2:**
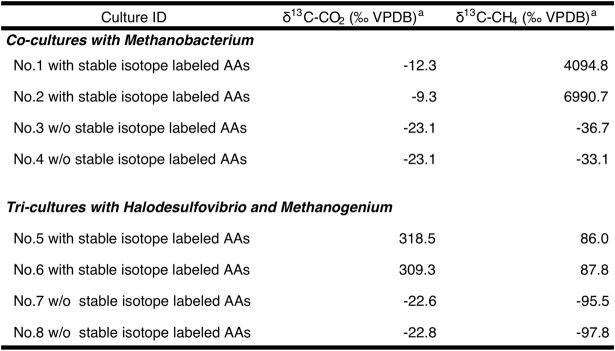
Carbon isotope fractionation values in MK-D1 cultures after 120 days incubation with and without stable isotope labelled amino acids

^a^Parts per thousand (‰) compared with the Vienna Pee Dee Belemnite (VPDB) standard.

**Extended Data Table 3:**
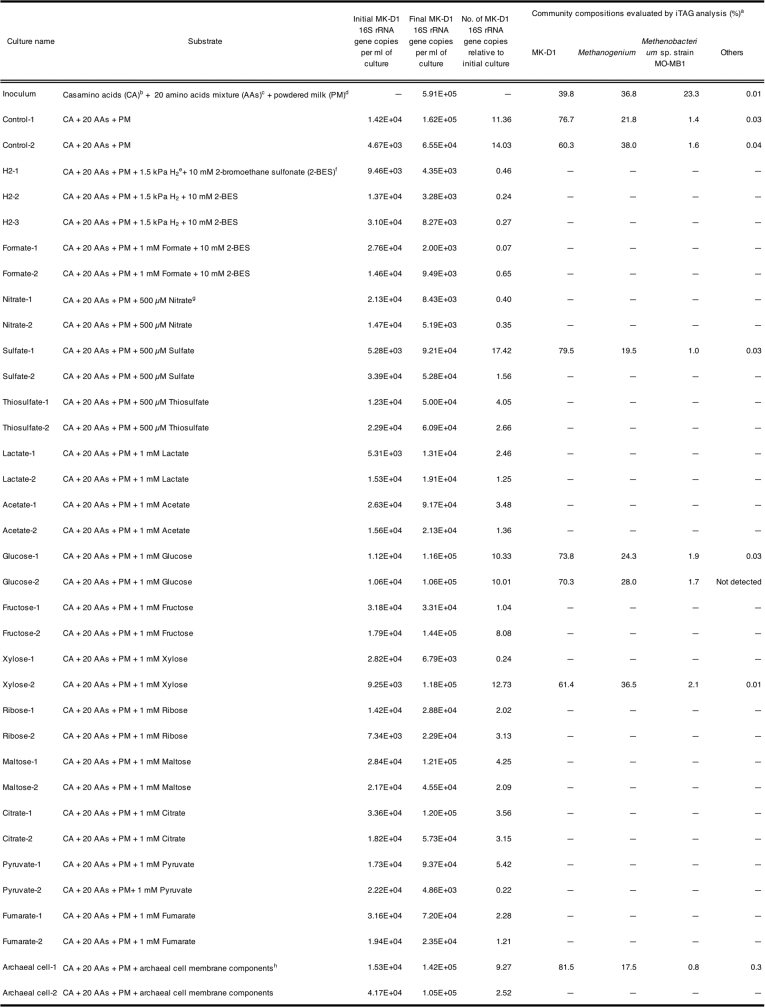
Growth of MK-D1 after incubation of 120 days with a range of substrates

A dash indicates that data were not taken for that sample.

^a^The iTAG analysis was performed for samples in which an increase of about 10 times or more in 16S rRNA gene copy numbers of MK-D1 was observed after incubation; data were analysed by qPCR assay. Detailed results are shown in Supplementary Table 1.

^b^Final concentration of casamino acids was 0.05% (w/v).

^c^Final concentration of each amino acid was 0.1 mM.

^d^Powdered milk for baby (Hohoemi, Meiji) was used at a final concentration of 0.1% (w/v).

^e^The concentration of hydrogen gas was in the head space of the culture bottle.

^f^2-BES was added to inhibit methanogens.

^g^Addition of nitrate completely suppressed the growth of MK-D1. This is probably because nitrate inhibits formate dehydrogenase activity of MK-D1^95^.

^h^Archaeal cell membrane components were a mixture of phytol, intact polar lipid–glycerol-dialkyl-glycerol tetraethers and core lipid– glycerol-dialkyl-glycerol tetraethers (each at a final concentration 50 ng ml^−1^). We used the archaeal membrane components as these have a positive effect on the growth of some archaeal species: (i) archaeal cell extract including membrane lipids stimulates the growth of the extremely thermophilic archaeon *Thermocaldium modestius*^96^, and (ii) the hyperthermophilic archaeon *Thermofilum pendes* requires the polar lipids for growth, which was obtained from the archaeal species *Thermoproteus tenax*^97^.

## Data Availability

Genomes for *Ca*. P. syntrophicum MK-D1, *Halodesulfovibrio* sp. MK-HDV and *Methanogenium* sp. MK-MG are available under GenBank BioProject accession numbers PRJNA557562, PRJNA557563 and PRJNA557565, respectively. The iTAG sequence data was deposited in BioProject PRJDB8518 with SRA accession numbers DRR184081–DRR184101. The 16S rRNA gene sequences of MK-D1, *Halodesulfovibrio* sp. MK-HDV, *Methanogenium* sp. MK-MG and clones obtained from primary enrichment culture were deposited in the DDBJ/EMBL/GenBank database under accession numbers LC490619–LC490624. The gene expression data of MK-D1 in BioProject PRJDB9032 with the accession number DRR199588. The cryo-electron tomograms of *Ca*. P. syntrophicum MK-D1 have been deposited in the EMDB with accession codes EMD-0809 and EMD-0852.

## References

[1] López-García P, Moreira D (2015). Open questions on the origin of eukaryotes. Trends Ecol. Evol..

[2] Martin WF, Garg S, Zimorski V (2015). Endosymbiotic theories for eukaryote origin. Phil. Trans. R. Soc. Lond. B.

[3] Eme L, Spang A, Lombard J, Stairs CW, Ettema TJG (2017). Archaea and the origin of eukaryotes. Nat. Rev. Microbiol..

[4] Koonin EV (2015). Origin of eukaryotes from within archaea, archaeal eukaryome and bursts of gene gain: eukaryogenesis just made easier? Phil. Trans. R. Soc. Lond. B.

[5] Spang A (2015). Complex archaea that bridge the gap between prokaryotes and eukaryotes. Nature.

[6] Zaremba-Niedzwiedzka K (2017). Asgard archaea illuminate the origin of eukaryotic cellular complexity. Nature.

[7] Sousa FL, Neukirchen S, Allen JF, Lane N, Martin WF (2016). Lokiarchaeon is hydrogen dependent. Nat. Microbiol..

[8] Seitz KW, Lazar CS, Hinrichs K-U, Teske AP, Baker BJ (2016). Genomic reconstruction of a novel, deeply branched sediment archaeal phylum with pathways for acetogenesis and sulfur reduction. ISME J..

[9] Dombrowski N, Teske AP, Baker BJ (2018). Expansive microbial metabolic versatility and biodiversity in dynamic Guaymas Basin hydrothermal sediments. Nat. Commun..

[10] Liu Y (2018). Comparative genomic inference suggests mixotrophic lifestyle for Thorarchaeota. ISME J..

[11] Seitz KW (2019). Asgard archaea capable of anaerobic hydrocarbon cycling. Nat. Commun..

[12] Spang A (2019). Proposal of the reverse flow model for the origin of the eukaryotic cell based on comparative analyses of Asgard archaeal metabolism. Nat. Microbiol..

[13] Pushkarev A (2018). A distinct abundant group of microbial rhodopsins discovered using functional metagenomics. Nature.

[14] Bulzu P-A (2019). Casting light on Asgardarchaeota metabolism in a sunlit microoxic niche. Nat. Microbiol..

[15] Aoki M (2014). A long-term cultivation of an anaerobic methane-oxidizing microbial community from deep-sea methane-seep sediment using a continuous-flow bioreactor. PLoS ONE.

[16] Schink, B. & Stams, A. J. in *The Prokaryotes: Prokaryotic Communities and Ecophysiology* (eds Rosenberg, E. et al.) 471–493 (Springer, 2013).

[17] Knittel K, Lösekann T, Boetius A, Kort R, Amann R (2005). Diversity and distribution of methanotrophic archaea at cold seeps. Appl. Environ. Microbiol..

[18] Albers S-V, Meyer BH (2011). The archaeal cell envelope. Nat. Rev. Microbiol..

[19] Marguet E (2013). Membrane vesicles, nanopods and/or nanotubes produced by hyperthermophilic archaea of the genus *Thermococcus*. Biochem. Soc. Trans..

[20] Rosenshine I, Tchelet R, Mevarech M (1989). The mechanism of DNA transfer in the mating system of an archaebacterium. Science.

[21] Imachi H (2011). Cultivation of methanogenic community from subseafloor sediments using a continuous-flow bioreactor. ISME J..

[22] Da Cunha V, Gaia M, Gadelle D, Nasir A, Forterre P (2017). Lokiarchaea are close relatives of Euryarchaeota, not bridging the gap between prokaryotes and eukaryotes. PLoS Genet..

[23] Da Cunha V, Gaia M, Nasir A, Forterre P (2018). Asgard archaea do not close the debate about the universal tree of life topology. PLoS Genet..

[24] Spang A (2018). Asgard archaea are the closest prokaryotic relatives of eukaryotes. PLoS Genet..

[25] Brunk CF, Martin WF (2019). Archaeal histone contributions to the origin of eukaryotes. Trends Microbiol..

[26] Buckel W, Thauer RK (2013). Energy conservation via electron bifurcating ferredoxin reduction and proton/Na^+^ translocating ferredoxin oxidation. Biochim. Biophys. Acta.

[27] Ma K, Zhou HZ, Adams MWW (1994). Hydrogen production from pyruvate by enzymes purified from the hyperthermophilic archaeon, *Pyrococcus furiosus*: a key role for NADPH. FEMS Microbiol. Lett..

[28] Nobu MK (2015). The genome of *Syntrophorhabdus aromaticivorans* strain UI provides new insights for syntrophic aromatic compound metabolism and electron flow. Environ. Microbiol..

[29] Martin W, Müller M (1998). The hydrogen hypothesis for the first eukaryote. Nature.

[30] Lyons TW, Reinhard CT, Planavsky NJ (2014). The rise of oxygen in Earth’s early ocean and atmosphere. Nature.

[31] Davín AA (2018). Gene transfers can date the tree of life. Nat. Ecol. Evol..

[32] Kump LR (2011). Isotopic evidence for massive oxidation of organic matter following the great oxidation event. Science.

[33] Andersson SG, Kurland CG (1999). Origins of mitochondria and hydrogenosomes. Curr. Opin. Microbiol..

[34] Fenchel T, Finlay BJ (1990). Oxygen toxicity, respiration and behavioural responses to oxygen in free-living anaerobic ciliates. J. Gen. Microbiol..

[35] Moreira D, López-García P (1998). Symbiosis between methanogenic archaea and δ-proteobacteria as the origin of eukaryotes: the syntrophic hypothesis. J. Mol. Evol..

[36] López-García P, Moreira D (2006). Selective forces for the origin of the eukaryotic nucleus. BioEssays.

[37] Burns JA, Pittis AA, Kim E (2018). Gene-based predictive models of trophic modes suggest Asgard archaea are not phagocytotic. Nat. Ecol. Evol..

[38] Martin WF, Tielens AGM, Mentel M, Garg SG, Gould SB (2017). The physiology of phagocytosis in the context of mitochondrial origin. Microbiol. Mol. Biol. Rev..

[39] Baum DA, Baum B (2014). An inside-out origin for the eukaryotic cell. BMC Biol..

[40] Hutson SM, Rannels SL (1985). Characterization of a mitochondrial transport system for branched chain α-keto acids. J. Biol. Chem..

[41] Hug LA, Stechmann A, Roger AJ (2010). Phylogenetic distributions and histories of proteins involved in anaerobic pyruvate metabolism in eukaryotes. Mol. Biol. Evol..

[42] Degli Esposti M (2016). Alpha proteobacterial ancestry of the [Fe–Fe]-hydrogenases in anaerobic eukaryotes. Biol. Direct.

[43] Pieulle L (1995). Isolation and characterization of the pyruvate-ferredoxin oxidoreductase from the sulfate-reducing bacterium *Desulfovibrio africanus*. Biochim. Biophys. Acta.

[44] Liebgott P-P (2010). Relating diffusion along the substrate tunnel and oxygen sensitivity in hydrogenase. Nat. Chem. Biol..

[45] Winkler HH, Neuhaus HE (1999). Non-mitochondrial ATP transport. Trends Biochem. Sci..

[46] Gray MW (2014). The pre-endosymbiont hypothesis: a new perspective on the origin and evolution of mitochondria. Cold Spring Harb. Perspect. Biol..

[47] Villanueva L, Schouten S, Damsté JSS (2017). Phylogenomic analysis of lipid biosynthetic genes of Archaea shed light on the ‘lipid divide’. Environ. Microbiol..

[48] Caforio A (2018). Converting *Escherichia coli* into an archaebacterium with a hybrid heterochiral membrane. Proc. Natl Acad. Sci. USA.

[49] Nakamura K (2006). Application of pseudomurein endoisopeptidase to fluorescence in situ hybridization of methanogens within the family *Methanobacteriaceae*. Appl. Environ. Microbiol..

[50] Cevc G, Richardsen H (1999). Lipid vesicles and membrane fusion. Adv. Drug Deliv. Rev..

[51] Nunoura T (2012). Microbial diversity in deep-sea methane seep sediments presented by SSU rRNA gene tag sequencing. Microbes Environ..

[52] Toki T, Higa R, Ijiri A, Tsunogai U, Ashi J (2014). Origin and transport of pore fluids in the Nankai accretionary prism inferred from chemical and isotopic compositions of pore water at cold seep sites off Kumano. Earth Planets Space.

[53] Nakahara N (2019). *Aggregatilinea lenta* gen. nov., sp. nov., a slow-growing, facultatively anaerobic bacterium isolated from subseafloor sediment, and proposal of the new order *Aggregatilineales* ord. nov. within the class *Anaerolineae* of the phylum *Chloroflexi*. Int. J. Syst. Evol. Microbiol..

[54] Murakami S, Fujishima K, Tomita M, Kanai A (2012). Metatranscriptomic analysis of microbes in an oceanfront deep-subsurface hot spring reveals novel small RNAs and type-specific tRNA degradation. Appl. Environ. Microbiol..

[55] Imachi H (2019). Cultivable microbial community in 2-km-deep, 20-million-year-old subseafloor coalbeds through ~1000 days anaerobic bioreactor cultivation. Sci. Rep..

[56] Miyashita A (2009). Development of 16S rRNA gene-targeted primers for detection of archaeal anaerobic methanotrophs (ANMEs). FEMS Microbiol. Lett..

[57] Yamaguchi T (2015). In situ DNA-hybridization chain reaction (HCR): a facilitated in situ HCR system for the detection of environmental microorganisms. Environ. Microbiol..

[58] Miyazaki M (2014). *Sphaerochaeta multiformis* sp. nov., an anaerobic, psychrophilic bacterium isolated from subseafloor sediment, and emended description of the genus *Sphaerochaeta*. Int. J. Syst. Evol. Microbiol..

[59] Toyooka K (2014). Wide-range high-resolution transmission electron microscopy reveals morphological and distributional changes of endomembrane compartments during log to stationary transition of growth phase in tobacco BY-2 cells. Plant Cell Physiol..

[60] Kremer JR, Mastronarde DN, McIntosh JR (1996). Computer visualization of three-dimensional image data using IMOD. J. Struct. Biol..

[61] Takano Y (2018). Insight into anaerobic methanotrophy from ^13^C/^12^C- amino acids and ^14^C/^12^C-ANME cells in seafloor microbial ecology. Sci. Rep..

[62] Okumura T (2016). Hydrogen and carbon isotope systematics in hydrogenotrophic methanogenesis under H_2_-limited and H_2_-enriched conditions: implications for the origin of methane and its isotopic diagnosis. Prog. Earth Planet. Sci..

[63] Takano Y, Kashiyama Y, Ogawa NO, Chikaraishi Y, Ohkouchi N (2010). Isolation and desalting with cation-exchange chromatography for compound-specific nitrogen isotope analysis of amino acids: application to biogeochemical samples. Rapid Commun. Mass Spectrom..

[64] Chikaraishi, Y. et al. *Instrumental Optimization for Compound-specific Nitrogen Isotope Analysis of Amino Acids by Gas Chromatography/Combustion/Isotope Ratio Mass Spectrometry in Earth, Life and Isotopes* (eds Ohkouchi, N. et al.) 367–386 (Kyoto Univ. Press, 2010). 10.1002/rcm.4651 20658677

[65] Leggett RM, Clavijo BJ, Clissold L, Clark MD, Caccamo M (2014). NextClip: an analysis and read preparation tool for Nextera long mate pair libraries. Bioinformatics.

[66] Bankevich A (2012). SPAdes: a new genome assembly algorithm and its applications to single-cell sequencing. J. Comput. Biol..

[67] Lin H-H, Liao Y-C (2016). Accurate binning of metagenomic contigs via automated clustering sequences using information of genomic signatures and marker genes. Sci. Rep..

[68] Boetzer M, Henkel CV, Jansen HJ, Butler D, Pirovano W (2011). Scaffolding pre-assembled contigs using SSPACE. Bioinformatics.

[69] Seemann T (2014). Prokka: rapid prokaryotic genome annotation. Bioinformatics.

[70] Marchler-Bauer A, Bryant SH (2004). CD-Search: protein domain annotations on the fly. Nucleic Acids Res..

[71] Marchler-Bauer A (2015). CDD: NCBI’s conserved domain database. Nucleic Acids Res..

[72] Jones P (2014). InterProScan 5: genome-scale protein function classification. Bioinformatics.

[73] Petersen TN, Brunak S, von Heijne G, Nielsen H (2011). SignalP 4.0: discriminating signal peptides from transmembrane regions. Nat. Methods.

[74] Yin Y (2012). dbCAN: a web resource for automated carbohydrate-active enzyme annotation. Nucleic Acids Res..

[75] Rawlings ND, Barrett AJ, Finn R (2016). Twenty years of the MEROPS database of proteolytic enzymes, their substrates and inhibitors. Nucleic Acids Res..

[76] Fischer M, Pleiss J (2003). The Lipase Engineering Database: a navigation and analysis tool for protein families. Nucleic Acids Res..

[77] Søndergaard D, Pedersen CNS, Greening C (2016). HydDB: a web tool for hydrogenase classification and analysis. Sci. Rep..

[78] Boutet E, Lieberherr D, Tognolli M, Schneider M, Bairoch A (2007). UniProtKB/Swiss-Prot. Methods Mol. Biol..

[79] Lima T (2009). HAMAP: a database of completely sequenced microbial proteome sets and manually curated microbial protein families in UniProtKB/Swiss-Prot. Nucleic Acids Res..

[80] Katoh K, Standley DM (2013). MAFFT multiple sequence alignment software version 7: improvements in performance and usability. Mol. Biol. Evol..

[81] Kozlov AM, Darriba D, Flouri T, Morel B, Stamatakis A (2019). RAxML-NG: a fast, scalable and user-friendly tool for maximum likelihood phylogenetic inference. Bioinformatics.

[82] Ronquist F (2012). MrBayes 3.2: efficient Bayesian phylogenetic inference and model choice across a large model space. Syst. Biol..

[83] Pruesse E, Peplies J, Glöckner FO (2012). SINA: accurate high-throughput multiple sequence alignment of ribosomal RNA genes. Bioinformatics.

[84] Quast C (2013). The SILVA ribosomal RNA gene database project: improved data processing and web-based tools. Nucleic Acids Res..

[85] Stamatakis A (2014). RAxML version 8: a tool for phylogenetic analysis and post-analysis of large phylogenies. Bioinformatics.

[86] Camacho C (2009). BLAST+: architecture and applications. BMC Bioinformatics.

[87] Fu L, Niu B, Zhu Z, Wu S, Li W (2012). CD-HIT: accelerated for clustering the next-generation sequencing data. Bioinformatics.

[88] UniProt Consortium (2019). UniProt: a worldwide hub of protein knowledge. Nucleic Acids Res..

[89] Edgar RC (2004). MUSCLE: multiple sequence alignment with high accuracy and high throughput. Nucleic Acids Res..

[90] Capella-Gutiérrez S, Silla-Martínez JM, Gabaldón T (2009). trimAl: a tool for automated alignment trimming in large-scale phylogenetic analyses. Bioinformatics.

[91] Guindon S (2010). New algorithms and methods to estimate maximum-likelihood phylogenies: assessing the performance of PhyML 3.0. Syst. Biol..

[92] Price MN, Dehal PS, Arkin AP (2010). FastTree 2—approximately maximum-likelihood trees for large alignments. PLoS ONE.

[93] Bolger AM, Lohse M, Usadel B (2014). Trimmomatic: a flexible trimmer for Illumina sequence data. Bioinformatics.

[94] Magoc T, Wood D, Salzberg SL (2013). EDGE-pro: estimated degree of gene expression in prokaryotic genomes. Evol. Bioinform. Online.

[95] Axley MJ, Grahame DA (1991). Kinetics for formate dehydrogenase of *Escherichia coli* formate-hydrogenlyase. J. Biol. Chem..

[96] Itoh T, Suzuki K, Nakase T (1998). *Thermocladium modestius* gen. nov., sp. nov., a new genus of rod-shaped, extremely thermophilic crenarchaeote. Int. J. Syst. Bacteriol..

[97] Zillig W (1983). The archaebacterium *Thermofilum pendens* represents, a novel genus of the thermophilic, anaerobic sulfur respiring *Thermoproteales*. Syst. Appl. Microbiol..

