# Supplementary material for: Isolation of an archaeon at the prokaryote–eukaryote interface
Source: Nature. 2020 Jan 15;577(7791):519–25. doi: 10.1038/s41586-019-1916-6 (PMC7015854; doi:10.1038/s41586-019-1916-6)
Supplement: Supplementary file 1 — This file contains Supplementary Notes 1–9, Supplementary Methods, Supplementary Figures 1–18, and Supplementary References. [file 41586_2019_1916_MOESM1_ESM.pdf]

nature

https://doi.org/10.1038/s41586-019-1916-6

Supplementary information

Isolation of an archaeon at the prokaryote–eukaryote interface

In the format provided by the authors and unedited

Hiroyuki Imachi, Masaru K. Nobu, Nozomi Nakahara, Yuki Morono, Miyuki Ogawara, Yoshihiro Takaki, Yoshinori Takano, Katsuyuki Uematsu, Tetsuro Ikuta, Motoo Ito, Yohei Matsui, Masayuki Miyazaki, Kazuyoshi Murata, Yumi Saito, Sanae Sakai, Chihong Song, Eiji Tasumi,

Yuko Yamanaka, Takashi Yamaguchi, Yoichi Kamagata, Hideyuki Tamaki & Ken Takai

Supplementary Information

This file contains Supplementary Notes 1–9, Supplementary Methods, Supplementary Figures 1–18, and Supplementary References.

Table of Contents

Supplementary Notes………02

Supplementary Methods…...07

Supplementary Figures…….11

Supplementary References...31

Supplementary Notes

Supplementary Note 1

Reason for using a continuous-flow bioreactor system to enrich deep marine sedimentary microorganisms and deep-sea methane seep sediment as an inoculum source. Culture-independent molecular studies showed that deep marine sediment harbors phylogenetically diverse microorganisms, most of which belong to uncultured taxa and are distinct from those living on the Earth's surface[1-4]. Hence, their physiology and metabolic functions still remain largely unknown[5,6]. To gain insight into deep marine sedimentary microbes, they need to be cultivated, and this has been a significant challenge. However, only a small fraction of indigenous deep subseafloor microbes has been successfully isolated and characterized[7,8]. It is unclear why the cultivation of deep marine sedimentary microbes is difficult, but the batch-type cultivation techniques commonly used in previous studies may have been inadequate for this purpose. Therefore, the development of a new cultivation technique is needed. We have, therefore, employed a continuous-flow bioreactor technique for the cultivation of deep marine sedimentary microbes since 2006. The bioreactor is called a down-flow hanging sponge (DHS) reactor, which was originally developed to treat municipal sewage at a low cost in developing countries[9-11]. Specifically, a polyurethane sponge packed in the DHS reactor column provides a large surface area for microbial colonization and a longer cell residence time. As such, this type of continuous-flow reactor cultivation can provide substrates at low concentrations, similarly to those found in the natural environment. In addition, continuous-flow bioreactors allow the outflow of metabolic products that may inhibit microbial growth if accumulated. These continuous-flow reactors thereby might increase the culturability of deep marine sedimentary microorganisms in a controlled manner and serve as better sources (incubators) for microbial isolation than the original samples[12]. In fact, using DHS reactors, we have successfully enriched phylogenetically diverse microorganisms from deep marine sediments[12-15] and isolated and characterized various microorganisms using enriched microbial community from the bioreactors[16-20].

In this study, we used deep-sea methane seep sediments collected off Kumano area, Japan. In deep-sea methane seep sediments, anaerobic oxidation of methane (AOM) reaction is the major microbial process and is mediated by a syntrophic association of euryarchaeal anaerobic methanotrophs (ANMEs) and deltaproteobacterial sulfate-

deducing bacteria (SRB)[21]. In addition to ANMEs and SRB, abundant and diverse microorganisms, most of which are affiliated with uncultured microbial groups of high taxonomic ranks, such as phylum, class, and order, live in methane-seep sediments[22-24]. Therefore, deep-sea methane-seep sediment can be regard as a hot spot for uncultured microorganisms. As such, cultivation and characterization of these uncultured microorganisms can greatly expand our knowledge regarding microbial physiology, genetics, and ecology. This was our rationale for using deep-sea methane seep sediment as an inoculum source for uncultured microorganism cultivation. However, in 2006, when we started the DHS bioreactor cultivation, there was extremely limited information about the metabolism of uncultured microorganisms because the metagenomic approach was not a common technique. We, therefore, could not predict the appropriate carbon and energy sources to culture uncultured microorganisms. However, we were aware that methane-seep microbial communities were sustained by methane released below the sea floor. Thus, we expected that if we provided methane as a major energy source in the DHS reactor system, the uncultured microorganisms could be cultivated from the methane-seep sediment under laboratory conditions, along with ANMEs and SRB. Indeed, using a combination of the DHS bioreactor "pre-enrichment" and subsequent *in vitro* cultivation, we have successfully cultured and isolated microorganisms representing predominant uncultured taxa. The "*Candidatus* Prometheoarchaeum syntrophicum" strain MK-D1 reported in this study is an example of cultured microorganism using our deep-sea methane seep-derived bioreactor enrichment.

Supplementary Note 2

Reason for use of the four antibiotics to isolate the Lokiarchaota. In parallel to the attempted the cultivation of microorganisms from methane-seep sediment, we tried to isolate anaerobic microorganisms from the enriched methanogenic microbial community in another DHS reactor, which was established from deep marine sediments collected off the Shimokita Peninsula, Japan[15]. During the isolation attempt, we detected few DSAG (i.e., Lokiarchaeota) sequences in a propionate-fed culture (Supplementary Table S8 in Imachi *et al.* [2011][15]). However, the Lokiarchaeota sequences became undetectable after five successive transfers. After this, we could not revive the culture any longer. However, we detected some Lokiarchaeota sequences in several anaerobic enrichment cultures supplemented with four antibiotics (i.e., ampicillin, vancomycin, kanamycin, and

streptomycin, each at a final concentration of 50 μg/ml), via archaeal 16S rRNA gene-based clone analysis (data not shown). This finding suggested that Lokiarchaeota members can tolerate these antibiotics. Therefore, we added these four antibiotics into the media to serve as selective agents for the isolation of Lokiarchaeota members from the enriched AOM microbial community in the DHS reactor.

Supplementary Note 3

Reason for provisional *Candidatus* status.

Although the strain was extensively characterized and other organisms have been isolated and published in co-cultures as well (*e.g.*, *Pelotomaculum schinkii* strain HH[25]), we opted to use the provisional *Candidatus* status due to challenges associated with (i) deposition and maintenance of the strain in culture collections (a requirement for validly naming a strain) stemming from the extremely low growth rate/yield and need for qPCR track growth and (ii) growing enough cell mass for accomplishing experiments that provide sufficient biological data for meeting the current standards for validly proposing a name for a strain.

Supplementary Note 4

Fate of ^{13}C-AAs in syntrophic degradation. MK-D1 and *Methanobacterium* co-cultures fed with ^{13}C-AAs generated ^{13}C-enriched CH_4 (Extended Data Table 2). On the other hand, ^{13}C-AA-fed tri-cultures of MK-D1 with *Halodesulfovibrio* and *Methanogenium* generated ^{13}C-enriched CO_2. Given that the medium is buffered with ^{12}C-HCO_3 and $^{12}CO_2$, any $^{13}CO_2$ produced by MK-D1 AA degradation would be diluted by the exogenous ^{12}C-carbonate pool and not directly reach the partner methanogen (*i.e.*, very little $^{13}CH_4$ generation); thus, syntrophy in the tri-culture is primarily mediated by the oxidative path. Syntrophy in the co-culture is likely mediated by the hydrolytic formate-transferring path as ^{13}C-AA-derived ^{13}C-formate would directly reach the symbiotic partner, enrich the intracellular carbonate pool through oxidation to $^{13}CO_2$, and supply $^{13}CO_2$ for $^{13}CH_4$ generation.

Supplementary Note 5

Abbreviations in Figure 4.

Monomeric FeFe hydrogenase (Hyd), trimeric electron-confurcating FeFe hydrogenase

(HydABC), reversible NADPH-dependent NiFe hydrogenase (HydAD), reversible heterodisulfide-dependent electron-confurcating hydrogenase (Mvh-Hdr), other NiFe hydrogenases (NiFe Hyd.), formate dehydrogenase (FdhA), putative electron-confurcating formate dehydrogenase (FdhA-HydBC), NADH-dependent NADPH:ferredoxin oxidoreductase (NfnAB), heterodisulfide- and flavin-dependent oxidoreductase of unknown function (FlxABCD), tetrahydromethanopterin methyltransferase (MT), Wood-Ljungdahl (WL), and methyl-CoM reductase (Mcr)

Supplementary Note 6

Potential benefits of syntrophic interaction with a sulfate-reducing bacterium.

With ocean oxygenation, sulfate levels rose[26], which may have afforded an opportunity for thermodynamically enhanced syntrophy via interaction with SRB[27] (an interaction evidenced by MK-D1 cultures). In addition, SRB are aerotolerant[28] and produce a reductant that can passively consume O_2 (H_2S). By contrast, the alternative syntrophic partner, methanogenic archaea, are sensitive to O_2 and produce an inert byproduct (CH_4). We suggest that interaction with SRB may have conferred a low-level of aerotolerance to enable the archaea to inhabit more oxygenated environments, where excess organic matter is predicted to have been available from increased cyanobacterial activity during the Great Oxidation Event[29,30]. Growth in oxygenated environments may have further selected for a more refined aerotolerance.

Supplementary Note 7

Transition towards aerobiosis may have benefited from multiple symbiosis.

To further adapt to higher O_2 concentrations and compete with facultatively aerobic organotrophs, interaction with an O_2-consuming partner might have been beneficial in gaining aerotolerance[31,32]. The alternative hypothesis would require the gain of genes for aerobic respiration, but this may have significantly weakened the selection for (endo)symbiosis between the host archaeon and pre-mitochondrial alphaproteobacterium symbiont (PA), assuming that the host-PA symbiosis that led to endosymbiosis was driven by the host's sensitivity to O_2 and dependency on an O_2-scavenging partner as we posit. Given that, with the current data, eukaryotes and *Ca.* Heimdallarchaeota represent the most recent branchpoint of archaea and eukaryotes, the lineages of *Ca.* Heimdallarchaeota that possess aerobic respiration genes[33] may have evolved along this

alternative route. Interaction with SRB may have also been beneficial in developing interaction with an O_2-utilizing partner. The ancestral Asgard archaeon is predicted to be an anaerobic AA-degrading H_2-producing organism dependent on partners for catabolic symbiosis (H_2/formate-scavenging; syntrophy) and anabolic (building block-supplying) symbioses. On the other hand, LECA was a facultatively aerobic organism that depended on mitochondria for O_2 respiration and ATP generation[34]. Given that no extant mitochondria (or related organelles) uptake H_2[35] (and reasons described in Supplementary Note 8), PA is suggested to have lacked ability to uptake H_2 and, thus, may not have been able to syntrophically support the host's catabolism. As we hypothesize the host may have required continued anaerobic catabolism (*i.e.*, gain ATP itself) during the transition towards aerobiosis until an ATP transport route from the endosymbiont to the host developed, the host may have benefited from continued syntrophic interaction with aerotolerant SRB.

Supplementary Note 8

Potential reason that H_2 transfer was unlikely to drive endosymbiosis.

Note that it is entirely possible for the H_2-consuming partner to have become endosymbionts of the pre-LECA archaeon as proposed previously[36,37]. However, we postulate that H_2-consumers would have had less advantage as endosymbionts. If the host gained an H_2-consuming endosymbiont, the host cell size may increase. In theory, compared to a smaller H_2-producing cell, an enlarged cell would stoichiometrically require a larger amount of substrate and energy (*i.e.*, through transport) to accumulate the same intracellular substrate concentration. Thus, to achieve the same intracellular H_2 concentration, more substrate and energy is hypothesized to be necessary for larger cells (*e.g.*, cells with an endosymbiont) as byproduct H_2 concentrations would stoichiometrically and thermodynamically depend on substrate concentrations. Moreover, H_2 is membrane-permeable, so, in terms of substrate transport, H_2 consumers gain little benefit from being inside an H_2 producer. Thus, endosymbiosis of an H_2 utilizer is unfavorable without uncompartmentalized H_2 generation and unlikely to have stabilized. In fact, extant H_2-consuming endosymbionts of eukaryotes (*i.e.*, some methanogenic archaea) are observed only in eukaryotes possessing compartmentalized H_2-producing organelles (*i.e.*, hydrogenosomes)[38]. Furthermore, based on current data, no extant mitochondria (or related organelles) can uptake H_2[35].

Supplementary Note 9

Potential impetus of developing mitochondrial ATP transport.

Our model assumes that the host evolved to delegate 2-oxoacid catabolism and ATP generation to the endosymbiont (supported by the interaction between extant eukaryotes and their mitochondria [and related organelles]), which suggests that an ATP transport mechanism would have been required to provide the host energy. Development of the ATP-providing machinery (*i.e.*, ATP:ADP carrier or AAC found in extant mitochondria) by the endosymbiont may have provided the opportunity for the host lose anaerobic O_2-sensitive 2-oxoacid catabolism (*i.e.*, 2-oxoacid:ferredoxin oxidoreductases and ferredoxin-dependent hydrogenases) to consequently lose catabolic O_2-sensitivity and delegate 2-oxoacid degradation and ATP synthesis to the endosymbiont. With current data, as the activity of AAC is reversible[39], it is unclear whether the endosymbiont developed AAC to altruistically transport ATP to the host or parasitically absorb ATP from the host (analogous to extant prokaryotes encoding ATP transporters – *Rickettsia* and *Chlamydiae*[37]). The above loss of anaerobic 2-oxoacid catabolism has potential evolutionary benefits for both scenarios. For the former, the above loss leads to consolidation of metabolism and delegation of catabolism to highly exergonic aerobic respiration. Interestingly, several theories point towards a parasitic origin of mitochondria[40–42]. If AAC was originally used for parasitism, the above loss of 2-oxoacid catabolism has the potential to resolve parasitism by reducing host ATP production and reversing the ATP transport direction of AAC (direction dependent on ATP concentration[39]).

Supplementary Methods

Culturing. The purity of *Ca*. P. syntrophicum strain MK-D1 was routinely examined by microscopy and iTAG analysis. In addition, the purity was verified by the whole genome shotgun sequencing, which only detected the sequences of MK-D1 and *Methanogenium* genomes. We also confirmed the culture purity based on failure to amplify the bacterial 16S rRNA gene through PCR using the bacterial primer pairs 27F/907R[24] and EUB338F*/1492R[43–45]. Moreover, we evaluated the culture purity based on the failure of microbial growth in the following media at 10°C, 20°C, 30°C, 37°C, and 55°C: (i) thioglycolate medium (Difco); (ii) basal medium supplemented with 1 mM sucrose, 1

mM glucose, 1 mM fructose, 1 mM xylose, and 0.01% (w/v) yeast extract; and (iii) basal medium containing 5 mM lactate, 10 mM sulfate, 0.05% (w/v) CA, and 0.01% (w/v) yeast extract.

To confirm *Halodesulfovibrio* has ability to use H_2 and formate, we isolated the bacterium from the MK-D1 enrichment culture using a roll-tube technique, with lactate (10 mM) and sulfate (10 mM), acting as an electron donor and acceptor, respectively. After isolation, we confirmed that the *Halodesulfovibrio*, designated strain MK-HDV, could grow on a hydrogen- or formate-fed medium supplemented with sulfate at 20°C.

Methanobacterium sp. strain MO-MB1 was previously isolated from subseafloor sediment in our laboratory as a hydrogen- and formate-utilizing methanogenic archaea[15]. *Methanobacterium* sp, strain MO-MB was cultured on the basal medium on the basal medium supplemented with H_2 (ca. 150 kPa in head space of culture bottle) and acetate (1 mM) and yeast extract (0.01%) at 30°C. For the SYBR Green I staining experiment (Extended Data Fig. 3m and 3n), MO-MB1 cells were fixed with 2% paraformaldehyde (PFA) after culturing under the condition mentioned above.

Methanogenium cariaci strain JR1 was obtained from the Japan Collection of Microorganisms (Tsukuba, Japan) and cultured on the basal medium supplemented with H_2 (ca. 150 kPa in head space of culture bottle) and acetate (1 mM) and yeast extract (0.01%) at 20°C.

Cultures of *Halodesulfovibrio* sp. strain MK-HDV and *Methanobacterium* sp. strain MO-MB1 have been deposited in Japan Collection for Microorganisms (JCM 32479 and JCM 18473, respectively).

Growth monitoring using qPCR. The reaction mixture for qPCR was prepared according to the manufacturer's protocol. To construct a template standard for the primer set, we used a dilution series of the 16S rRNA gene amplicon of MK-D1, which was obtained via clone analysis using an archaeal primer pair Arch21F/1492R[46,47]. The dilution series of the PCR product was used in each qPCR analysis to calculate the 16S rRNA gene copy number. Template DNA was quantified using a Quant-iT dsDNA High-Sensitivity Assay Kit (Life Technologies). The PCR condition was as follows: initial denaturation at 95°C for 30 s, followed by 40 cycles of denaturation at 95°C for 10 s, annealing at 58°C for 30 s, and extension at 72°C for 31 s. The annealing temperature was optimized empirically through the amplification of the 16S rRNA gene of MK-D1. To verify the specificity of the qPCR assay, we performed two types of experiments. First,

a melting-curve analysis was performed for every qPCR assay. Second, the PCR product size was confirmed by gel electrophoresis and subsequent clone library analysis. The clone library experiment was performed only for three DNA samples, which were obtained from two- and three-successive transferred Lokiarchaeota enrichment cultures and the DHS bioreactor enrichment. We confirmed that all the PCR products showed the expected PCR amplicon size (i.e., about 390 bp) and all retrieved clones were identical to MK-D1 or were affiliated with the candidate phylum Lokiarchaeota (16 clones were randomly collected from each library).

FISH. MK-D1-specific probes were designed using the probe design tool of the ARB program[48]. The specificity of the probes was confirmed using the BLAST and the ARB-SILVA databases[49]. The $\Delta G^{0}_{overall}$ values of the probes and target MK-D1 16S rRNA sequence were calculated using the mathFISH web server[50]. Both probes exhibited high hybridization efficiencies (-15.5 kcal/mol for DSAG-Gr2-1142, -14.4 kcal/mol for DSAG-Gr2-1432).

Growth test using multiple substrates. A highly purified culture of MK-D1 was inoculated in the medium (15% inoculum, v/v). Then, 1 ml of the culture liquid was immediately taken from each culture to examine the initial 16S rRNA gene copy numbers of MK-D1 using the qPCR technique. The liquid culture samples for qPCR analysis were stored at -80°C until further processing. After the sampling, all the cultures were incubated at 20°C for 120 days in the dark without shaking. After 120 days of incubation, 1 ml of liquid culture was sampled from each culture vial to quantify the final 16S rRNA gene copy numbers of MK-D1. DNA extraction and qPCR analysis were performed, as the methods mentioned in the Methods section. In the qPCR assay, samples taken from the same culture vial (e.g., 0-day and 120-day samples of H2-1 culture) were applied to the same PCR plate for accurate quantification. To confirm microbial community structure after incubation, iTAG analysis was performed on the samples, which showed an increase of about 10-fold or more in the 16S rRNA gene copy numbers of MK-D1 after incubation, as observed by the qPCR assay.

Stable isotope probing incubation and NanoSIMS analysis. During incubation, 5 ml of culture liquid was taken from the vials once every 30 days, 1 ml of which was used for qPCR and iTAG analyses, and the remaining 4 ml was processed for NanoSIMS analysis. The samples for NanoSIMS analysis were fixed in 2% PFA under anaerobic condition for approximately 2 h. The fixed cells were washed twice in PBS and stored in a 1:1 mixture

of PBS and ethanol at -20°C until further processing.

Due to the low-biomass sample, the fixed cells were concentrated in a small analysis area (0.5–1 mm^2) of indium tin oxide-coated polycarbonate membranes using a fluorescence-activated cell sorting[51]. Microbial cells on membranes were stained with SYBR Green I and observed with an epifluorescence microscope (BX-51, Olympus) prior to NanoSIMS analysis.

Samples were analyzed by raster ion imaging with a CAMECA NanoSIMS 50L at the Kochi Institute for Core Sample Research, JAMSTEC. A focused primary Cs^+ beam of ~1 pA for carbon and nitrogen isotopic analysis was rastered over 30 × 30 μm^2 areas on the samples. Negative secondary ions of ^{12}C (EM#1), ^{13}C (EM#2), ^{16}O (EM#4), $^{12}C^{14}N$ (EM#5), $^{12}C^{15}N$ (EM#6), and ^{32}S (EM#7) were measured using six electron multipliers (EMs) in a multi-detection mode at a high mass resolving power of ~7,000 (CAMECA NanoSIMS definition), which is sufficient to separate all relevant isobaric interferences (i.e., ^{13}C on $^{12}C^{1}H$, $^{12}C^{14}N$ on $^{13}C^{++}$, and $^{12}C^{14}N^{1}H$). Each run was initiated after the stabilization of the secondary ion beam intensity following a pre-sputtering of approximately 2 min with a strong primary ion beam current. The same area was repeatedly scanned (20–30 times) in each run, with individual images consisting of 256 × 256 pixels and with a dwell time of 2,000 μs. The total acquisition time was approximately 1 h.

The recorded images and data were analyzed using IDL based NASA JSC imaging software for NanoSIMS[52], OpenMIMS (https://github.com/BWHCNI/OpenMIMS) and Look@NanoSIMS[53]. The images were corrected for quasi-simultaneous arrival effect and detector dead time. Different scans of each image were aligned to correct image drift during acquisition. The final images were generated by adding the secondary ion counts of each recorded secondary ion from each pixel for all scans.

Supplementary Figures

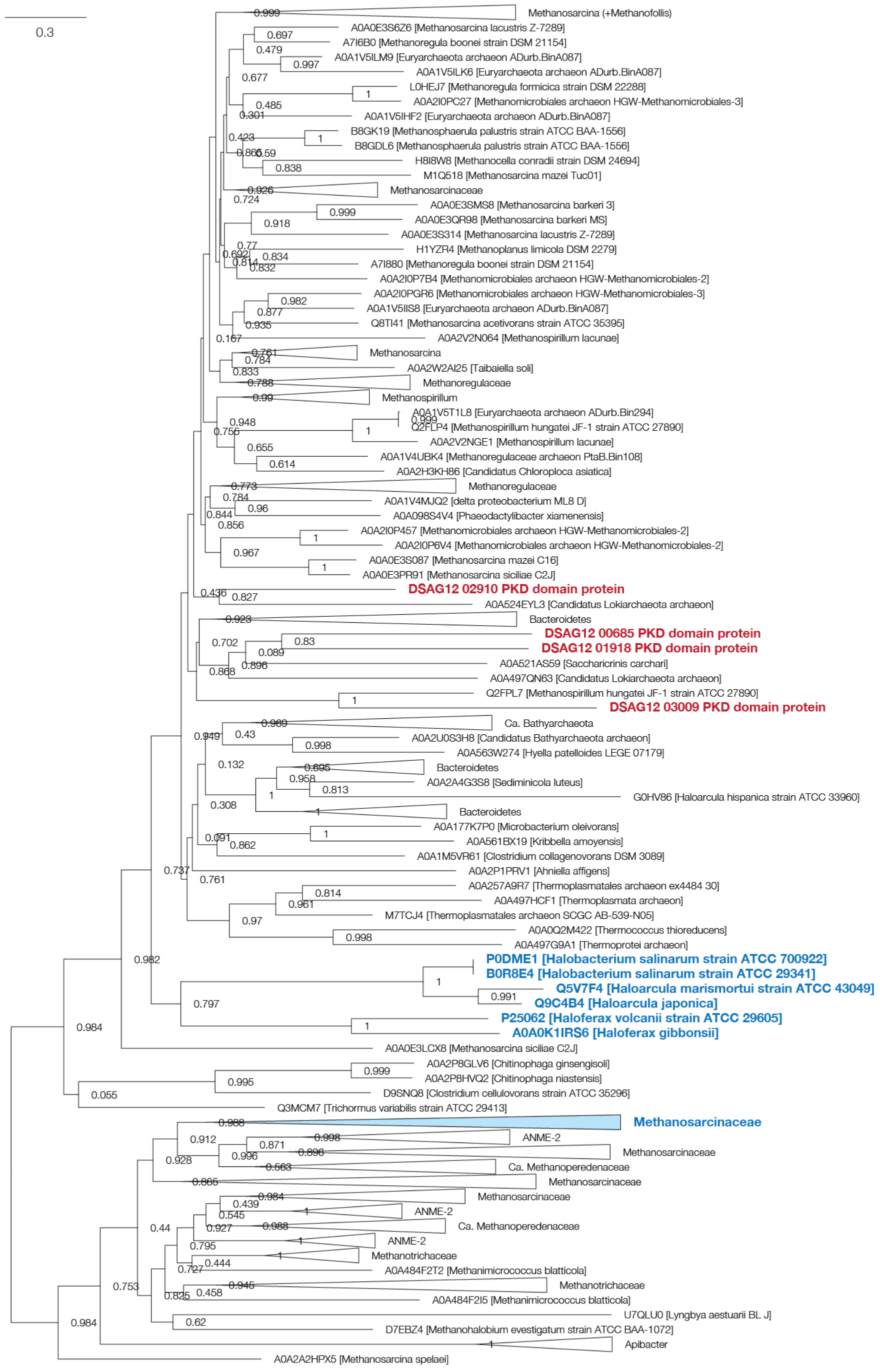

Supplementary Figure 1 | Phylogenetic tree of S-layer proteins related to MK-D1 and *Euryarchaeota*. Putative S-layer proteins of MK-D1 (PKD domain proteins) were compared with related homologs (identified by blastp against the UniProt database 2019_09 (UniProt Consortium NAR 2019 10.1093/nar/gky1049) and only kept hits with similarity ≥20% and alignment overlap ≥40%) and *Methanosarcina barkeri* S-layer protein (UniProt ID: Q46BP2) homologs (same as above). Sequences were clustered with a 70% similarity cutoff using CD-HIT v4.8.1[54] and aligned with MAFFTv7[55]. Positions with gaps in ≥30% of the sequences were trimmed with trimAl v1.2 (-gt 0.7)[56], leaving

429 sites total. The tree was constructed using FastTree[57] (-lg) with 1000 bootstrap replicates.

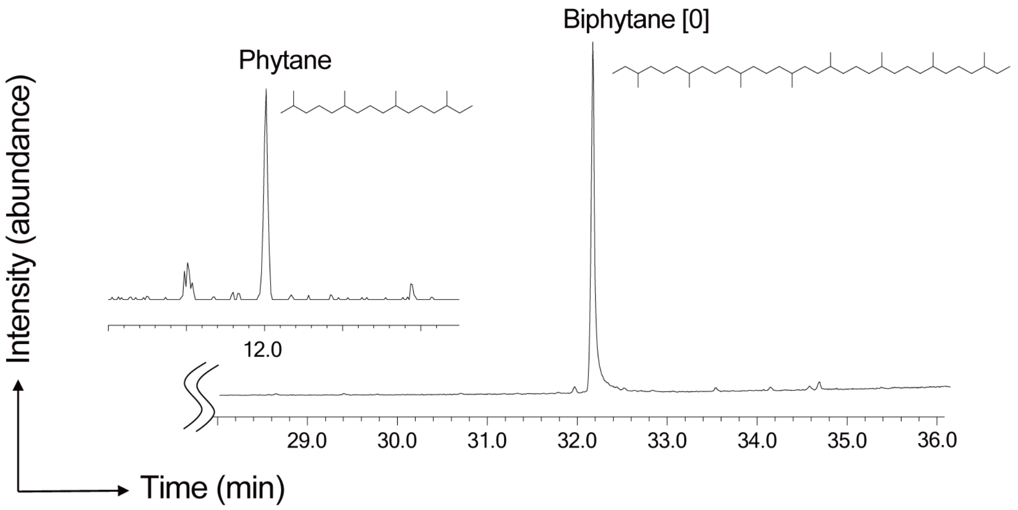

Supplementary Figure 2 | A total ion chromatogram of gas chromatography/mass spectrometry (GC/MS) for lipid obtained from *Methanogenium cariac*i strain JR1 (JCM 10550). The chemical structures of isoprenoid lipids are also shown.

Supplementary Figure 3 | Hypothetical ESCRT-III-related proteins with Snf7 domain. **a**, Phylogenetic tree of putative Snf7 domain proteins identified in the genome of strain MK-D1 (red), homologs from the UniProt 2019_06 database (≥20% similarity and ≥60% coverage based on blastp, representatives selected with CD-Hit [70% similarity]), and eukaryotic Snf7 family proteins verified experimentally (blue). The maximum likelihood tree was constructed using a MAFFT sequence alignment, trimming positions with gaps in ≥50% of sequences (232 positions remaining), and RAxML-NG[58] (--model LG+G4+F; 100 bootstrap replicates). **b**, Domain analysis based on sequences registered in NCBI CDD (v3.17) Snf7 domain pfam03357. A sequence logo is shown for the region of the CDD alignment overlapping with the consensus sequence registered in CDD. AA colors are based on chemistry (polar, neutral, basic, acidic, and hydrophobic). For each position, the letter width corresponds to the percentage of non-gap sequences. MK-D1 sequences were aligned to the CDD reference alignment (mafft --add x --keeplength). Residues aligned with any positions with bit values ≥2 are bolded and colored based on chemistry. Those with chemistry consistent with at least one residue in the CDD alignment are shown with a yellow background (otherwise orange background).

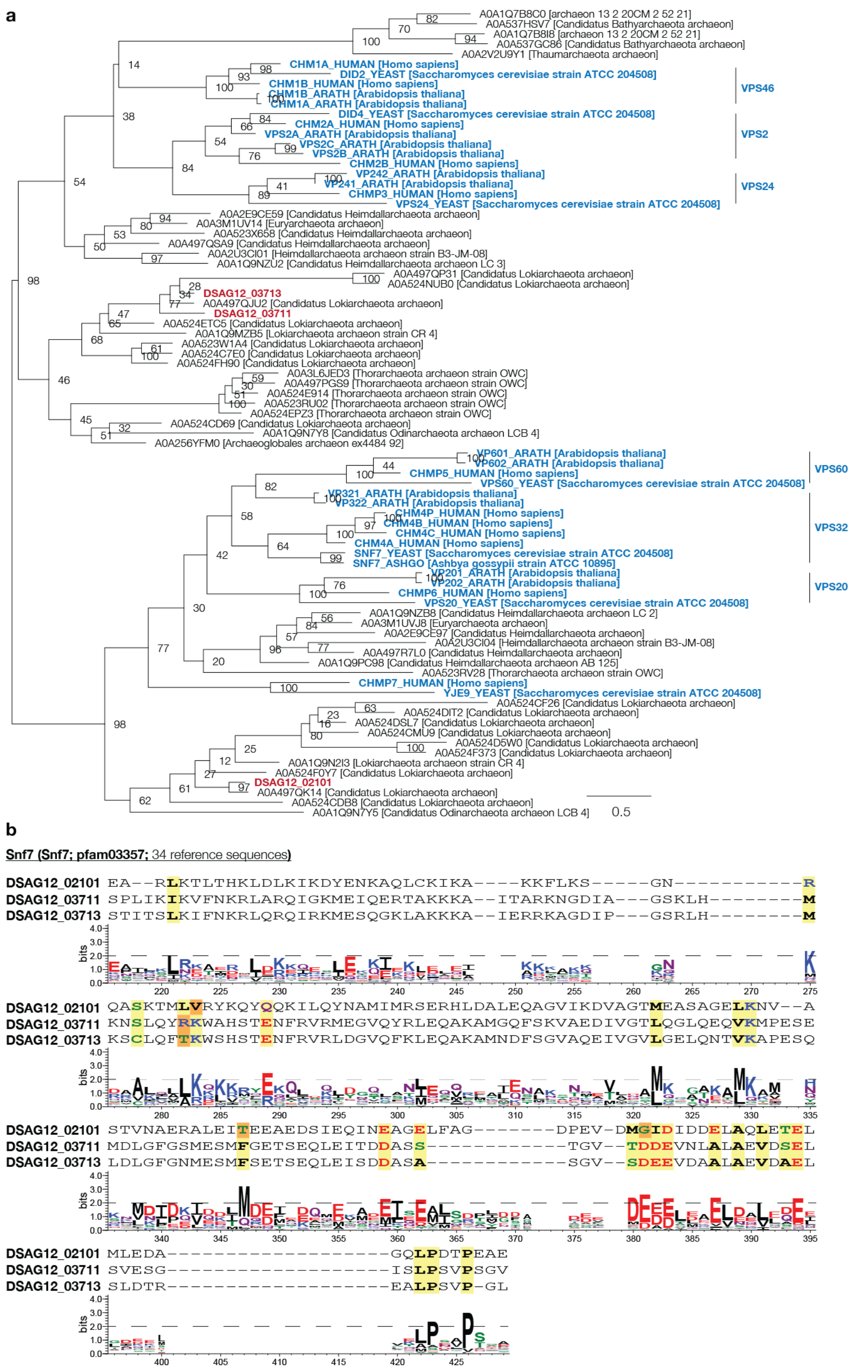

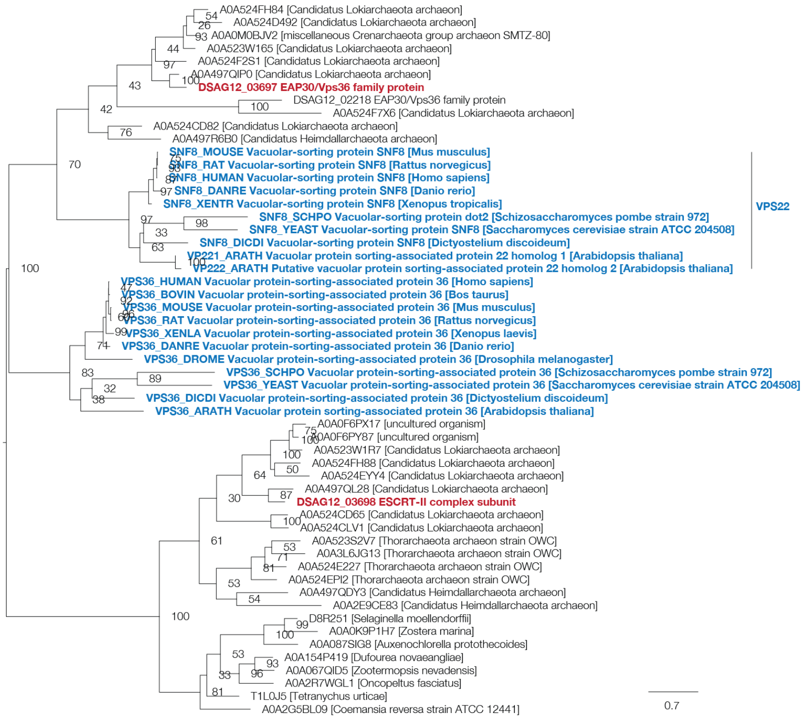

Supplementary Figure 4 | Hypothetical ESCRT-II-related proteins with EAP30/Vps36-related domain. Phylogenetic tree of putative EAP30/Vps36 domain proteins identified in the genome of strain MK-D1 (red), homologs from the UniProt 2019_06 database (≥20% similarity and ≥60% coverage based on blastp, representatives selected with CD-Hit [80% similarity]), and eukaryotic EAP30/Vps26 family proteins verified experimentally (blue). The maximum likelihood tree was constructed using a MAFFT-based sequence alignment (1096 positions), and RAxML-NG (--model LG+G4+F; 100 bootstrap replicates).

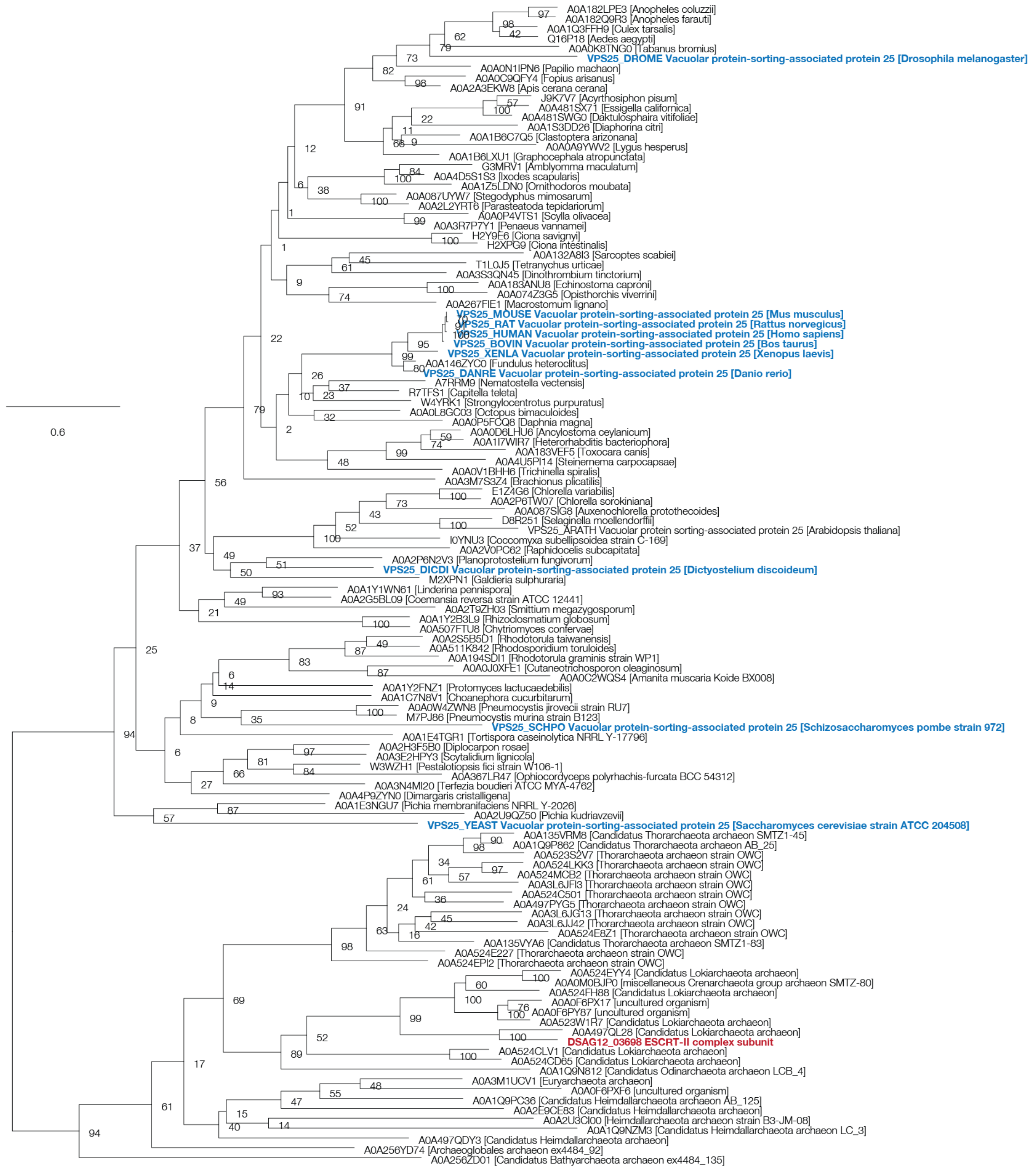

Supplementary Figure 5 | Hypothetical ESCRT-II-related proteins with Vps25-related domain. Phylogenetic tree of putative ESCRT II domain proteins identified in the genome of strain MK-D1 (red), homologs from the UniProt 2019_06 database (≥25% similarity and ≥60% coverage based on blastp, representatives selected with CD-Hit [80% similarity]), and eukaryotic Vps25 family proteins verified experimentally (blue). The maximum likelihood tree was constructed using a MAFFT-based sequence alignment (2305 positions), and RAxML-NG (--model LG+G4+F; 100 bootstrap replicates).

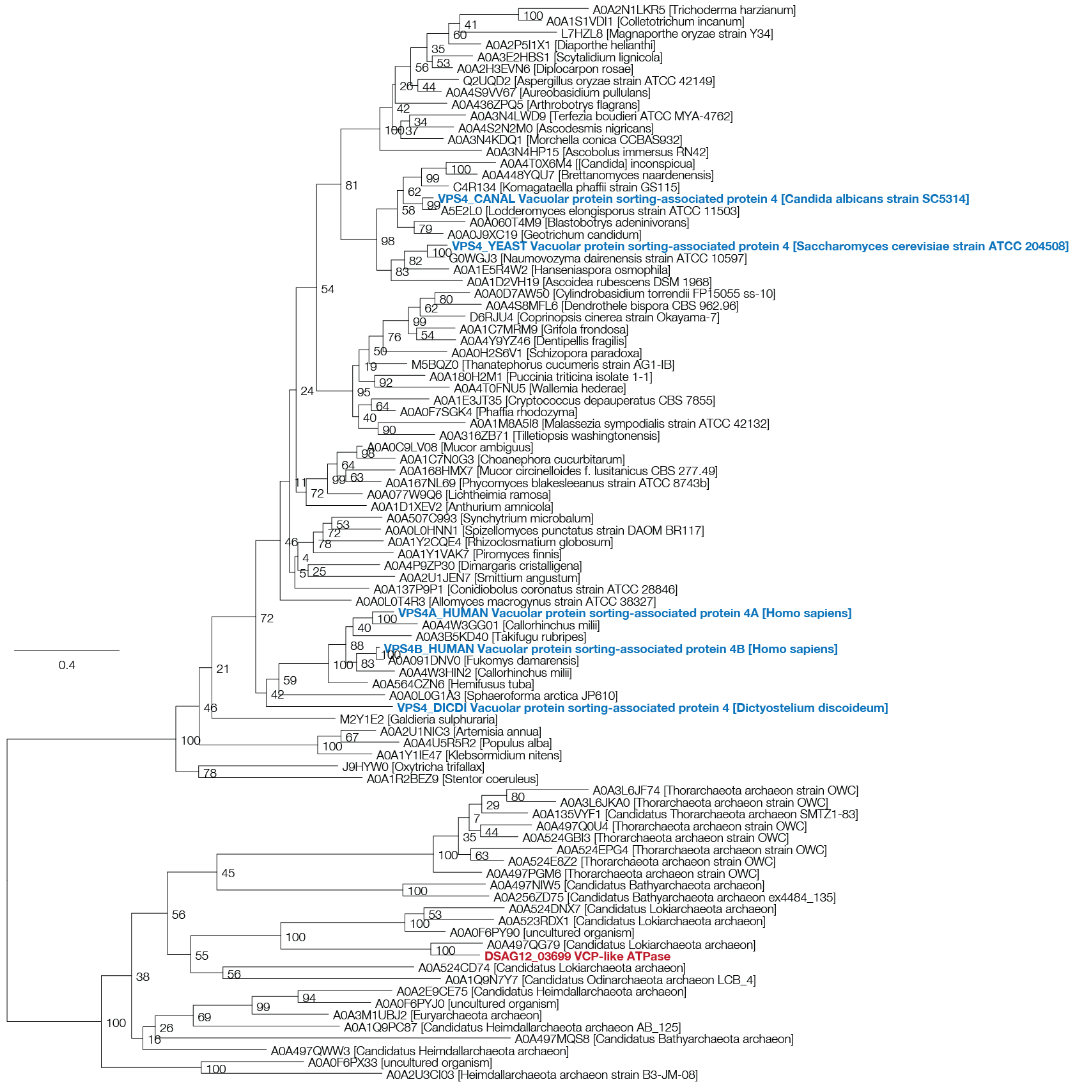

Supplementary Figure 6 | Hypothetical Vps4 ATPase-related proteins with Vps4-related domain. Phylogenetic tree of putative Vps4 domain proteins identified in the genome of strain MK-D1 (red), homologs from the UniProt 2019_06 database (≥30% similarity and ≥60% coverage based on blastp, representatives selected with CD-Hit [80% similarity]), and eukaryotic Vps4 family proteins verified experimentally (blue). The maximum likelihood tree was constructed using a MAFFT-based sequence alignment (2305 positions), and RAxML-NG (--model LG+G4+F; 100 bootstrap replicates).

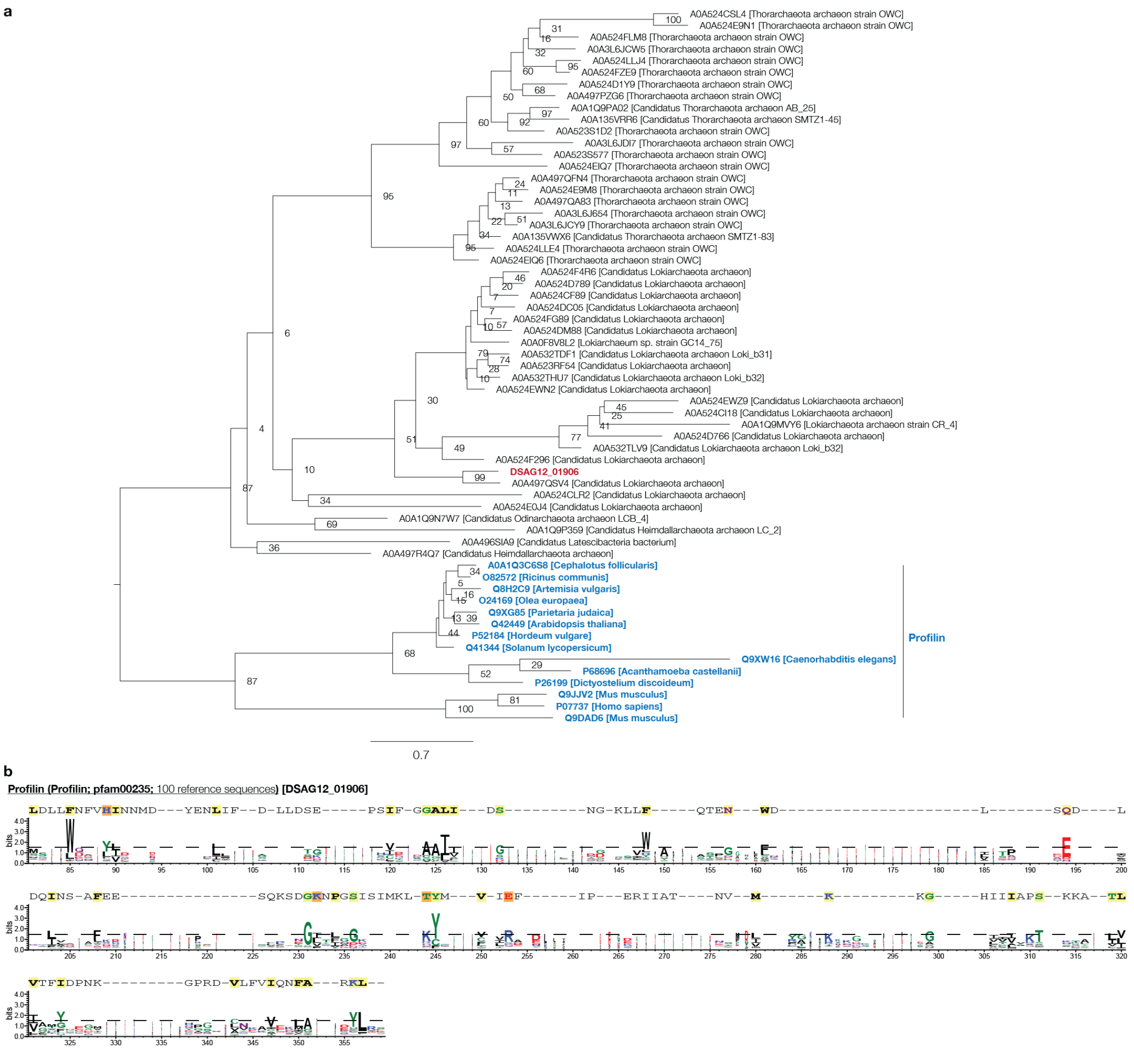

Supplementary Figure 7 | Hypothetical protein with profilin-like domain. a, Maximum likelihood tree. Homologs were collected through BLASTp analysis of the Asgard archaea sequences against the UniProt database (release 2019_09). Of homologs with sequence similarity ≥20%, representative sequences were selected using CD-HIT (-c 0.8). Additional homologs with verified biochemical activity, sequence similarity ≥20% were collected through BLASTp analysis of the Asgard archaea sequences against the UniProt/SwissProt database (2019_09). Sequences were aligned using MAFFT v7. Only sequences with ≥70% overlap with the corresponding MK-D1 sequence were retained for further analyses. The phylogenetic tree was constructed using RAxML-NG (--model LG+G4+F) and 100 bootstrap replicates. 221 sites of the alignment were used for tree construction. **b**, Domain analysis based on sequences registered in NCBI CDD (v3.17) profilin domain pfam00235. A sequence logo is shown for the region of the CDD alignment overlapping with the consensus sequence registered in CDD. Amino acid colors are based on their chemistry (polar, neutral, basic, acidic, and hydrophobic). For each position, the letter width corresponds to the percentage of non-gap sequences. MK-D1 sequences were aligned to the CDD reference alignment (mafft --add x --keeplength). Residues aligned with any positions with bit values ≥1.5 are bolded and colored based on their chemistry. Those with chemistry consistent with at least one residue in the CDD alignment are shown with a yellow background (otherwise orange background).

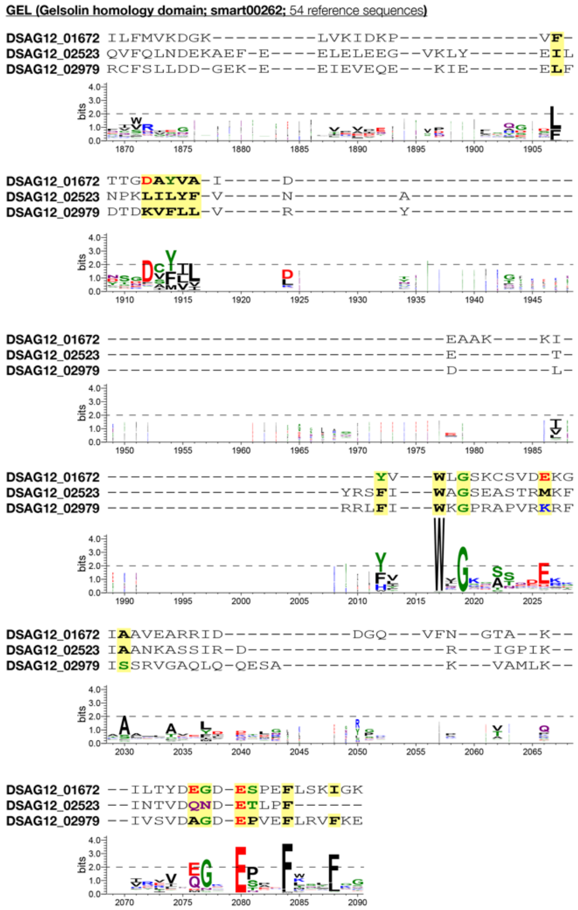

Supplementary Figure 8 | Hypothetical proteins with gelsolin-like domains. Domain analysis based on sequences registered in NCBI CDD (v3.17) gelsolin domain smart00262. A sequence logo is shown for the region of the CDD alignment overlapping with the consensus sequence registered in CDD. Amino acid colors are based on their chemistry (polar, neutral, basic, acidic, and hydrophobic). For each position, the letter width corresponds to the percentage of non-gap sequences. MK-D1 sequences were aligned to the CDD reference alignment (mafft --add x --keeplength). Residues aligned with any positions with bit values ≥2 are bolded and colored based on their chemistry. Those with chemistry consistent with at least one residue in the CDD alignment are shown with a yellow background (otherwise orange background).

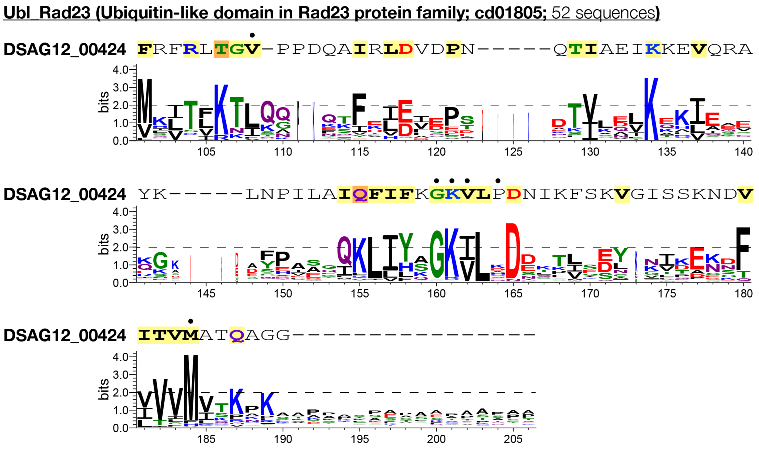

Supplementary Figure 9 | Hypothetical protein with ubiquitin-like domain. Domain analysis based on sequences registered in NCBI CDD (v3.17) ubiquitin-like domain cd01805. A sequence logo is shown for the region of the CDD alignment overlapping with the consensus sequence registered in CDD. Amino acid colors are based on their chemistry (polar, neutral, basic, acidic, and hydrophobic). For each position, the letter width corresponds to the percentage of non-gap sequences. MK-D1 sequences were aligned to the CDD reference alignment (mafft --add x --keeplength). Residues aligned with any positions with bit values ≥2 are bolded and colored based on their chemistry. Those with chemistry consistent with at least one residue in the CDD alignment are shown with a yellow background (otherwise orange background). Positions with catalytic functions (documented in CDD) are marked with black dots.

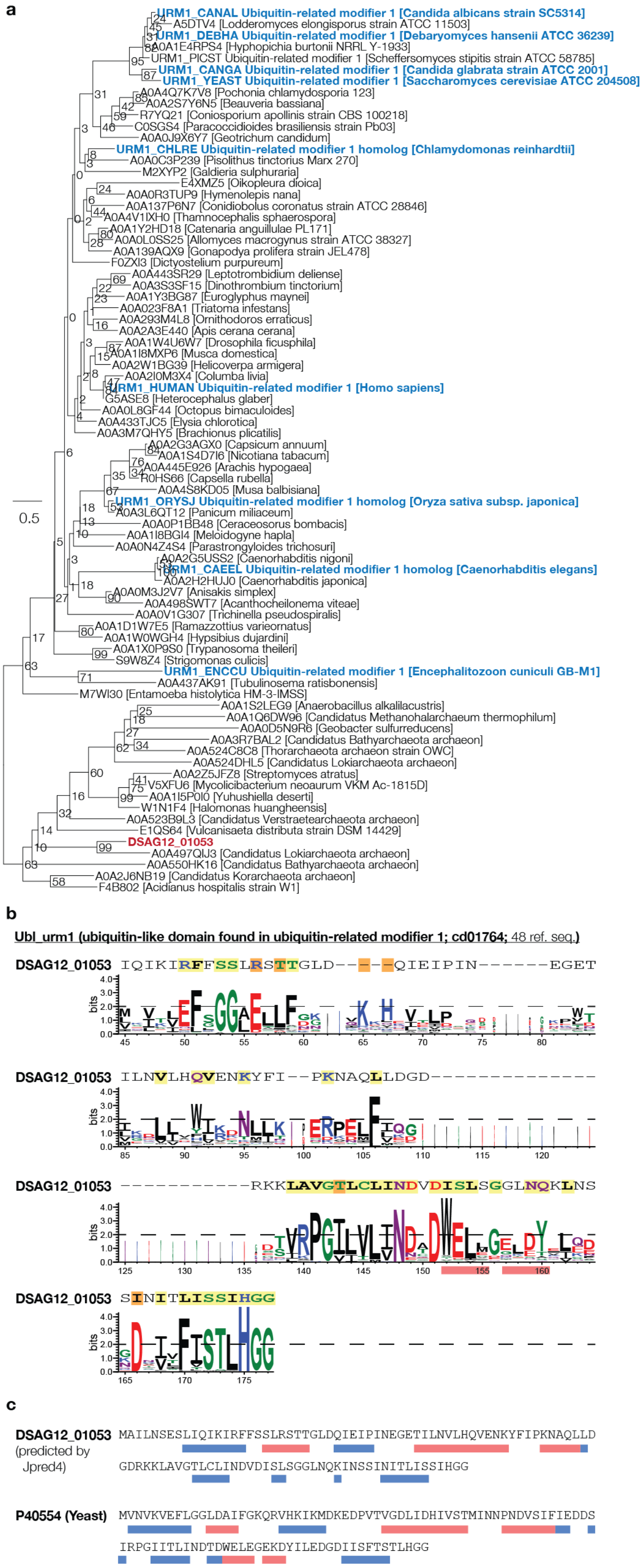

Supplementary Figure 10 | Hypothetical protein with ubiquitin-related domain. a, Phylogenetic tree of putative ubiquitin-related modifier domain proteins identified in the genome of strain MK-D1 (red), homologs from the UniProt 2019_06 database (≥20% similarity and ≥60% coverage based on blastp, representatives selected with CD-Hit [70% similarity]), and eukaryotic ubiquitin-related modifiers verified experimentally (blue). The maximum likelihood tree was constructed using a MAFFT sequence alignment (417 positions), and RAxML-ng (--model LG+G4+F; 100 bootstrap replicates). **b**, Domain analysis based on sequences registered in NCBI CDD (v3.17) ubiquitin-related modifier domain pfam03357. A sequence logo is cd01764 for the region of the CDD alignment overlapping with the consensus sequence registered in CDD. Amino acid colors are based on their chemistry (polar, neutral, basic, acidic, and hydrophobic). For each position, the letter width corresponds to the percentage of non-gap sequences. MK-D1 sequences were aligned to the CDD reference alignment (mafft --add x --keeplength). Residues aligned with any positions with bit values ≥2 are bolded and colored based on their chemistry. Those with chemistry consistent with at least one residue in the CDD alignment are shown with a yellow background (otherwise orange background). **c**, Comparison of secondary structure between eukaryotic ubiquitin-related modifier and MK-D1 homolog (Jpred4-predicted) (blue = beta sheet; red = alpha helix).

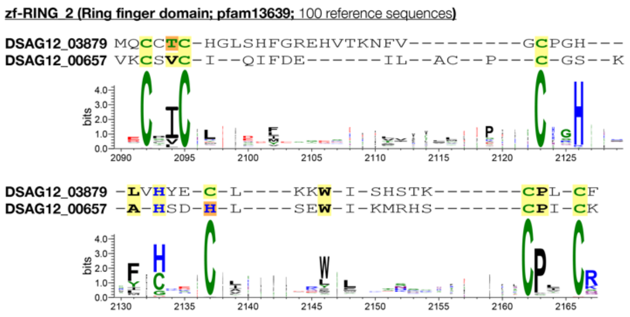

Supplementary Figure 11 | Hypothetical protein with RING finger-like domain. Domain analysis based on sequences registered in NCBI CDD (v3.17) RING finger domain pfam13639. A sequence logo is shown for the region of the CDD alignment overlapping with the consensus sequence registered in CDD. Amino acid colors are based on their chemistry (polar, neutral, basic, acidic, and hydrophobic). For each position, the letter width corresponds to the percentage of non-gap sequences. MK-D1 sequences were aligned to the CDD reference alignment (mafft --add x --keeplength). Residues aligned with any positions with bit values ≥2 are bolded and colored based on their chemistry. Those with chemistry consistent with at least one residue in the CDD alignment are shown with a yellow background (otherwise orange background).

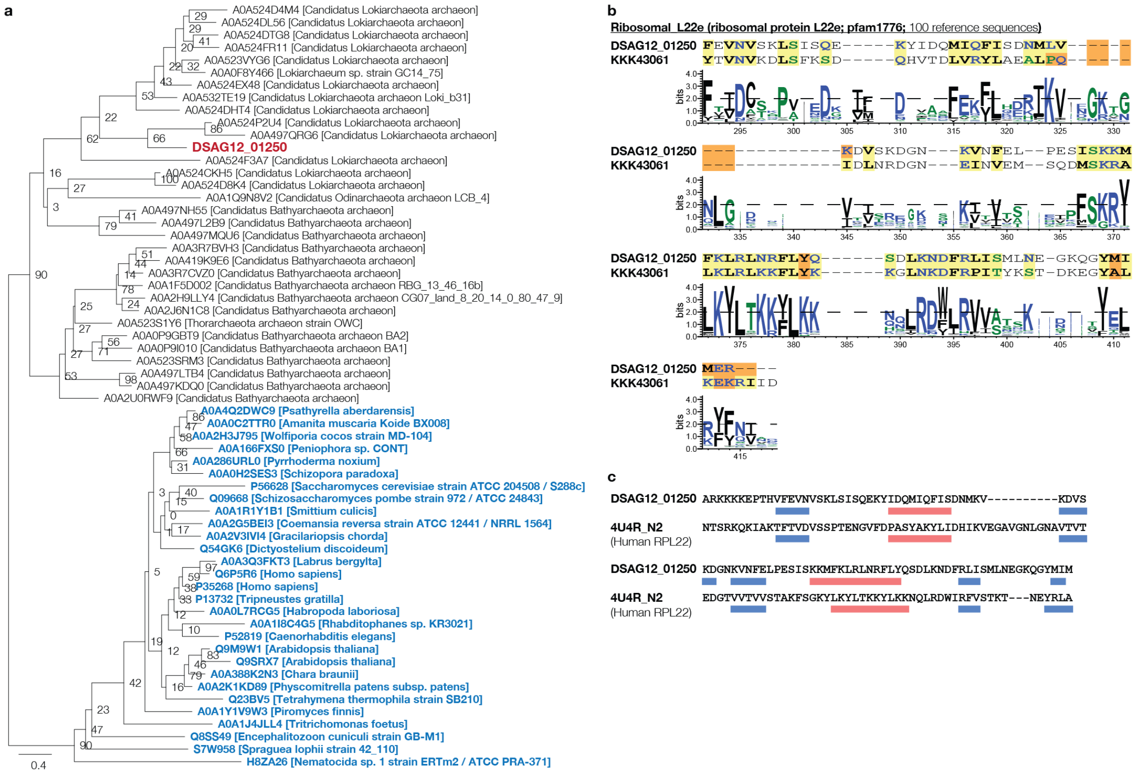

Supplementary Figure 12 | Putative ribosomal protein L22e. a, Maximum likelihood tree of genes encoding putative ribosomal protein L22e. Homologs were collected through BLASTp analysis of the Asgard archaea sequences against the UniProt database (release 2019_09). Of homologs with sequence similarity ≥20%, representative sequences were selected using CD-HIT with a clustering cutoff of 80% similarity (default settings otherwise). Additional homologs with verified biochemical activity, sequence similarity ≥20% were collected through BLASTp analysis of the Asgard archaea sequences against the UniProt/SwissProt database (2019_09). Sequences were aligned using MAFFT v7 with default settings. Only sequences with ≥70% overlap with the corresponding MK-D1 sequence were retained for further analyses. The phylogenetic tree was constructed using RAxML-NG using fixed empirical substitution matrix (LG), 4 discrete GAMMA categories, empirical amino acid frequencies from the alignment, and 100 bootstrap replicates. 225 sites of the alignment were used for tree construction. **b**, Domain analysis based on sequences registered in NCBI CDD (v3.17) ribosomal protein L22e pfam1776. A sequence logo is shown for the region of the CDD alignment overlapping with the consensus sequence registered in CDD. Amino acid colors are based on their hydrophobicity (hydrophilic, neutral, and hydrophobic). For each position, the letter width corresponds to the percentage of non-gap sequences. MK-D1 sequences were aligned to the CDD reference alignment (mafft --add x --keeplength). Residues aligned with any positions with bit values ≥2 are bolded and colored based on their chemistry. Those with chemistry consistent with at least one residue in the CDD alignment are shown with a yellow background (otherwise orange background). **c**, Comparison of secondary structure between eukaryotic ribosomal protein L22e and MK-D1 homolog (HHpred-predicted) (blue = beta sheet; red = alpha helix).

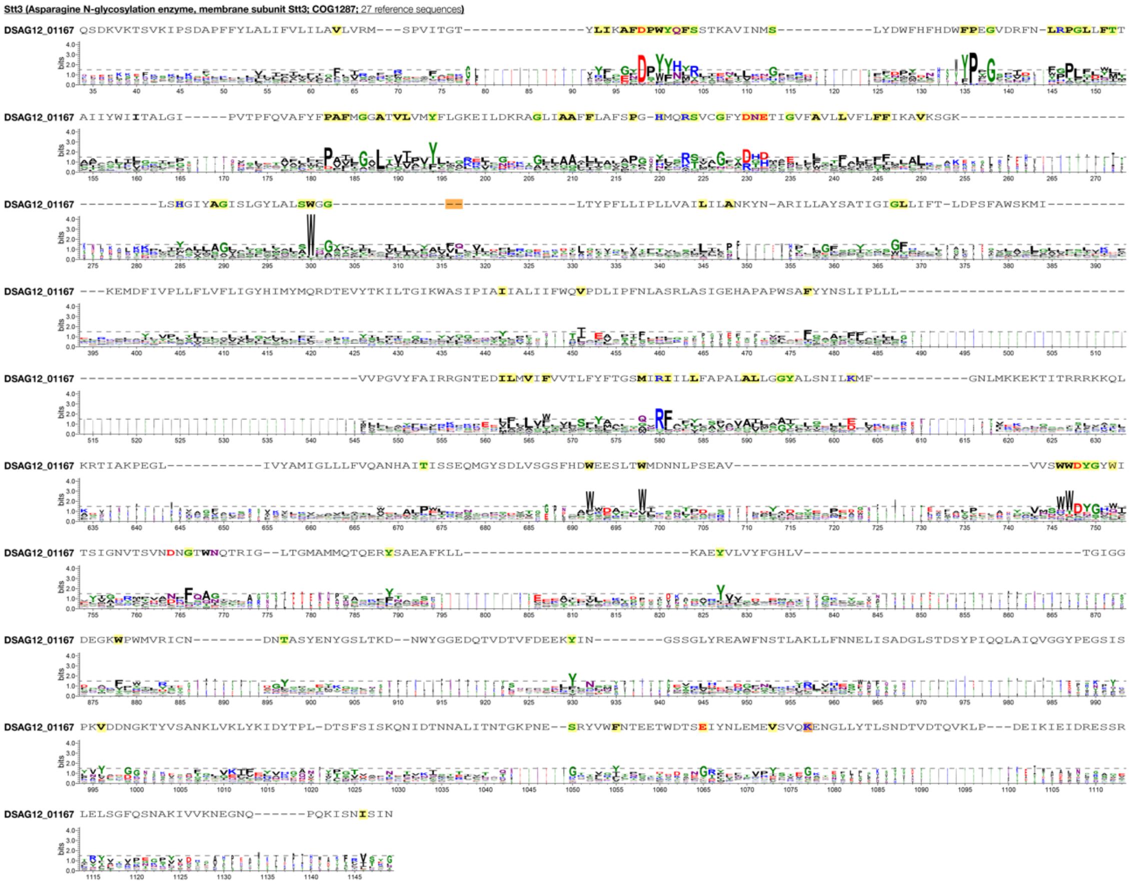

Supplementary Figure 13 | Hypothetical protein with N-glycosylation enzyme membrane subunit Stt3-like domain. Domain analysis based on sequences registered in NCBI CDD (v3.17) N-glycosylation enzyme membrane subunit Stt3 domain COG1287. A sequence logo is shown for the region of the CDD alignment overlapping with the consensus sequence registered in CDD. Amino acid colors are based on their chemistry (polar, neutral, basic, acidic, and hydrophobic). For each position, the letter width corresponds to the percentage of non-gap sequences. MK-D1 sequences were aligned to the CDD reference alignment (mafft --add x --keeplength). Residues aligned with any positions with bit values ≥1.5 are bolded and colored based on their chemistry. Those with chemistry consistent with at least one residue in the CDD alignment are shown with a yellow background (otherwise orange background).

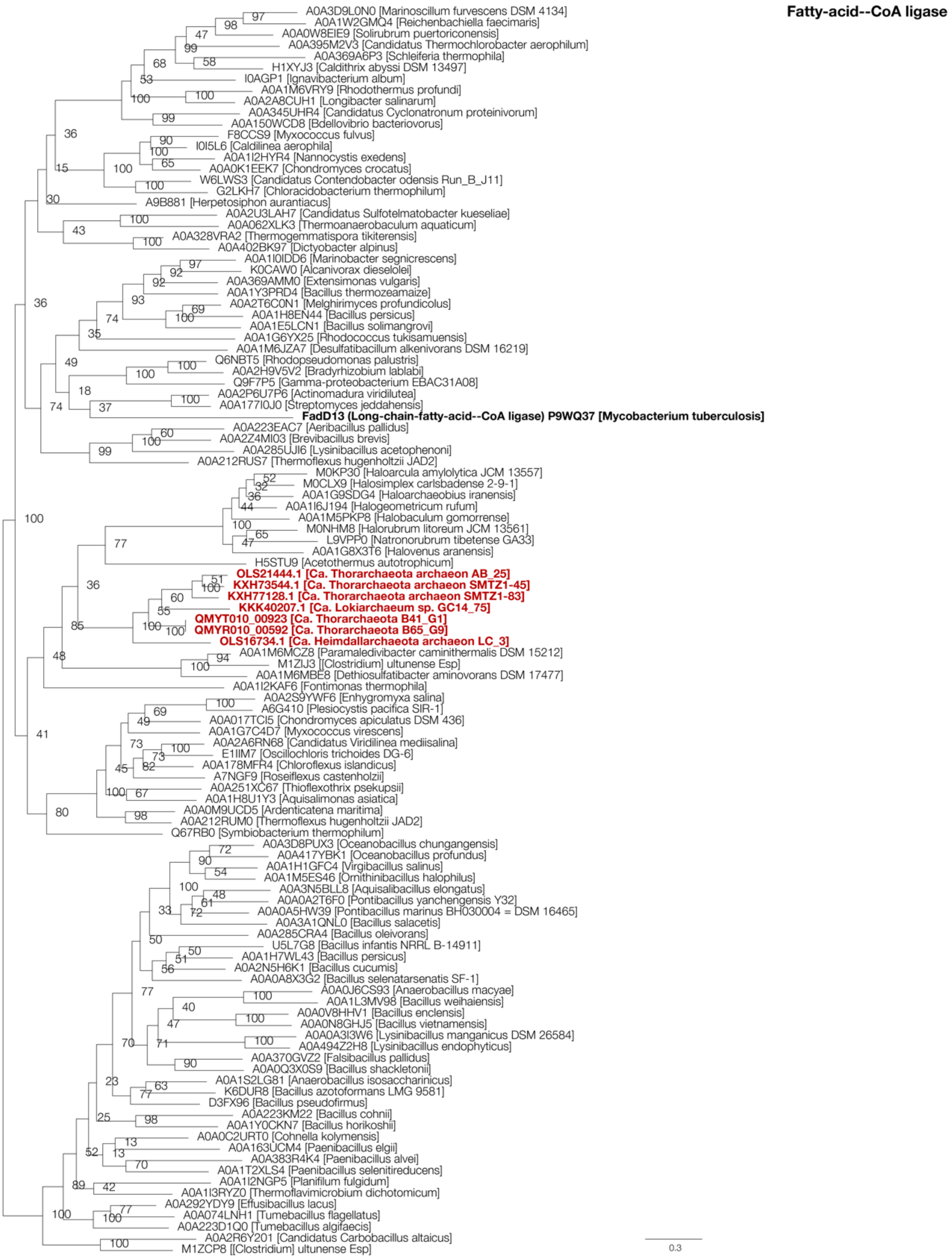

Supplementary Figure 14 | Maximum likelihood tree of Asgard archaea fatty-acid--CoA ligase. Although the closest characterized relative of the Asgard archaea genes is a long-chain-fatty-acid--CoA ligase, the preferred substrate remains unclear as fatty-acid--CoA ligases have broad/diverse substrate specificities. See Extended Data Figure 6 caption for details. 831 sites of the alignment were used for tree construction.

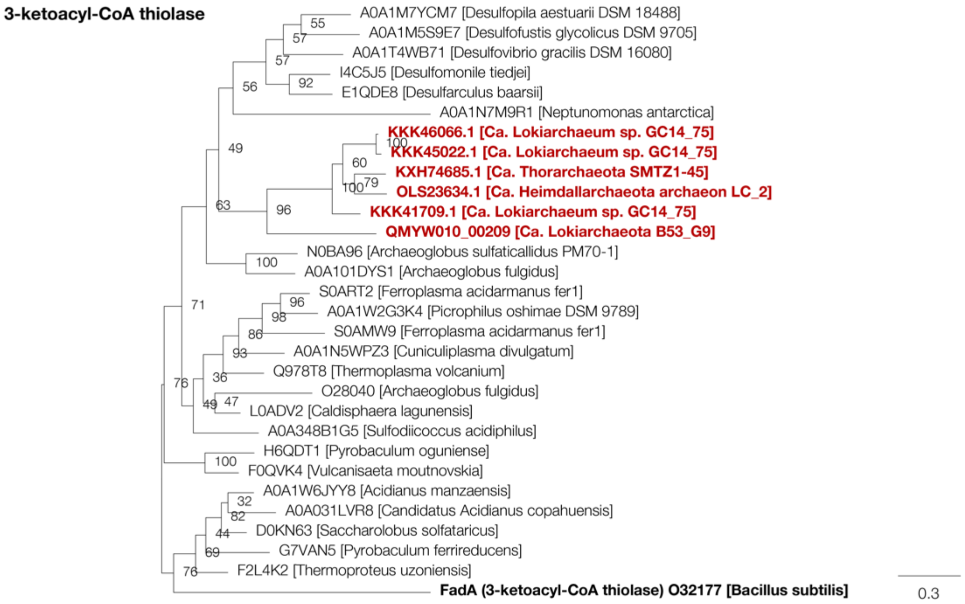

Supplementary Figure 15 | Maximum likelihood tree of Asgard archaea 3-ketoacyl-CoA thiolase (FadA). See Extended Data Figure 6 caption for details. 489 sites of the alignment were used for tree construction.

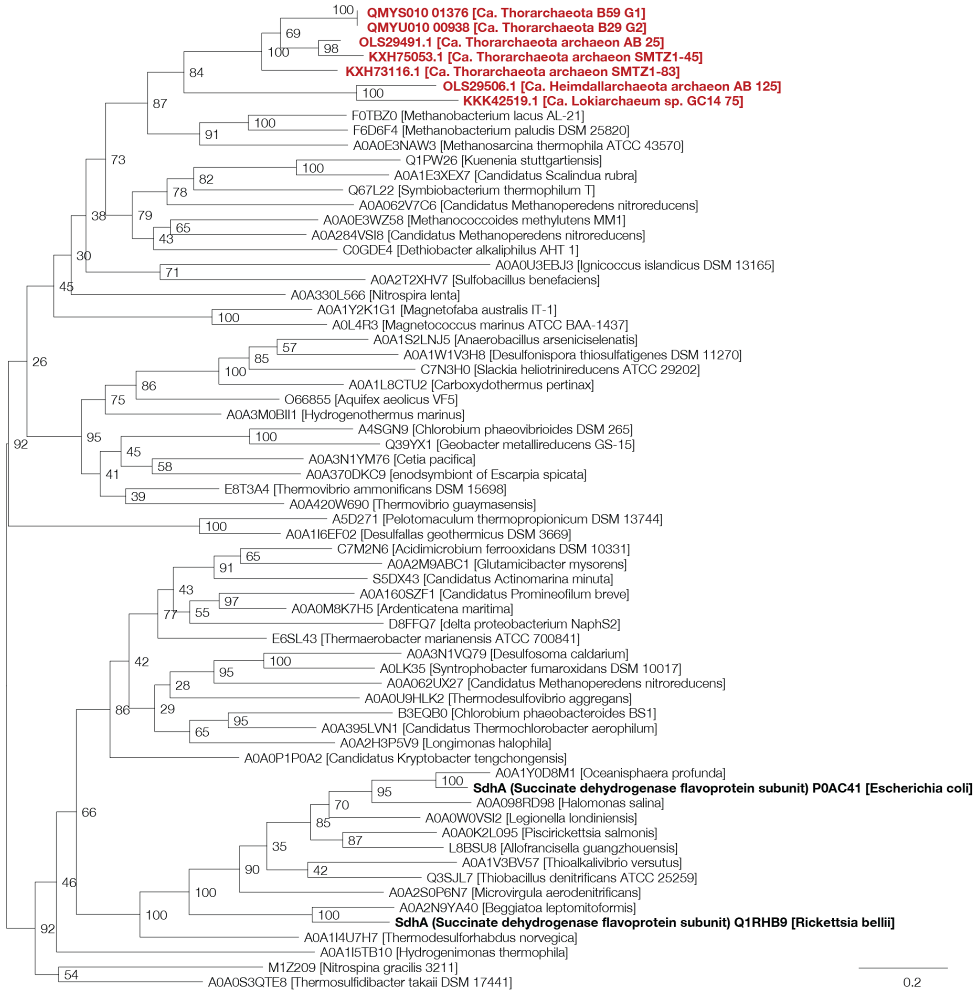

Supplementary Figure 16 | Maximum likelihood tree of Asgard archaea succinate dehydrogenase flavoprotein subunit (SdhA). See Extended Data Figure 6 caption for details. MUSCLE v.3.8.31 was used for alignment instead of MAFFT and CD-HIT clustering was performed with a 60% cutoff rather than 70%. Positions with gaps in more than 10% of the sequences were excluded from the alignment using trimAl v1.2 (-gt 0.9; and default settings otherwise). 553 sites of the alignment were used for tree construction.

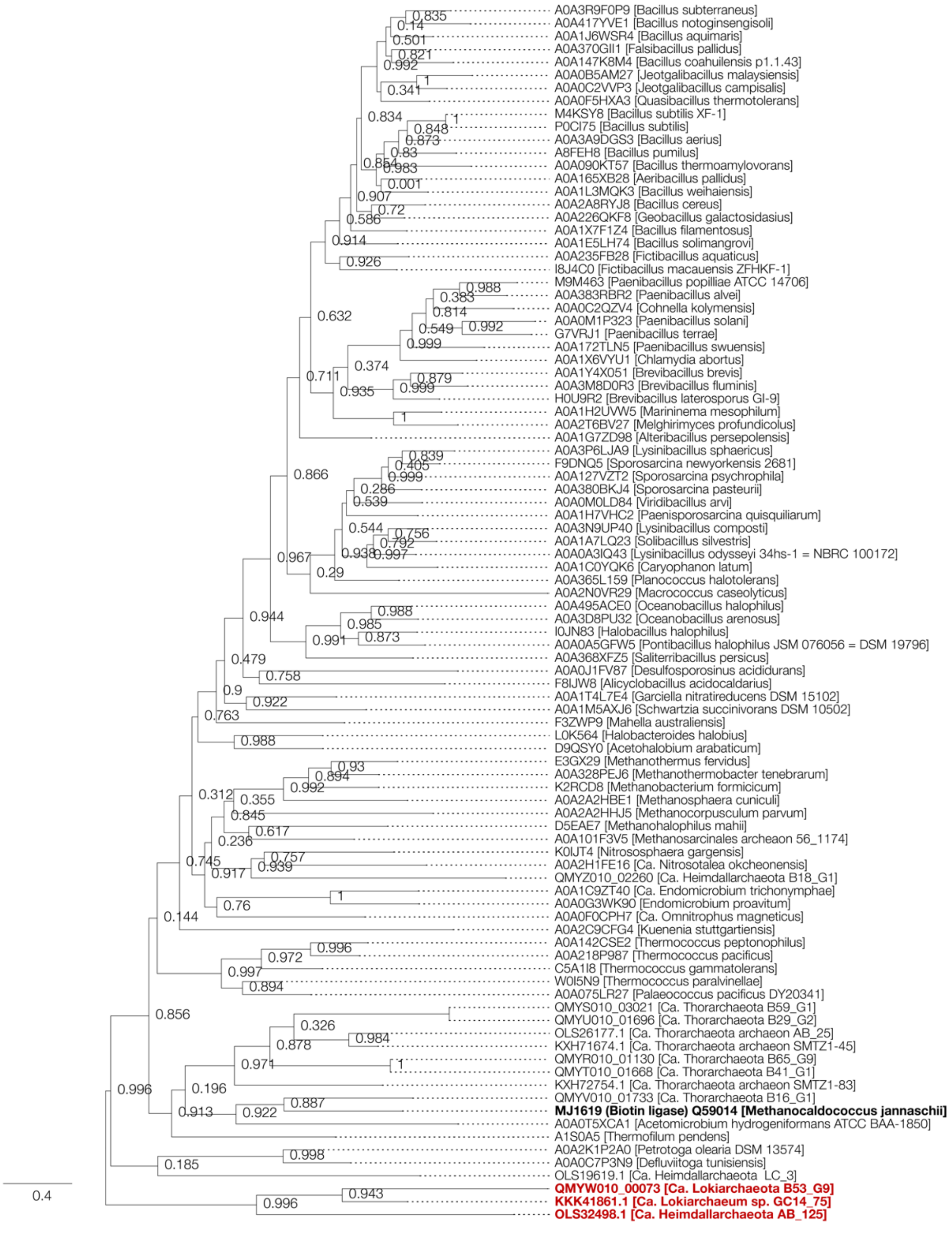

Supplementary Figure 17 | Maximum likelihood tree of Asgard archaea biotin ligase (BirA). BirA homologs were collected through BLASTp analysis of the Asgard archaea sequences against the UniProt database (release 2019_06). Of homologs with sequence similarity ≥40% and overlap ≥70%, representative sequences were selected using CD-HIT with a clustering cutoff of 70% similarity (default settings otherwise). Additional homologs with verified biochemical activity, sequence similarity ≥30%, and overlap ≥70% were collected through BLASTp analysis of the Asgard archaea sequences against the UniProt/SwissProt database. Sequences were aligned using MAFFT v7 with default settings and trimmed using trimAl with default settings. The phylogenetic tree was

constructed using FastTree using fixed empirical substitution matrix (LG) and 1000 bootstrap replicates. 332 sites of the alignment were used for tree construction.

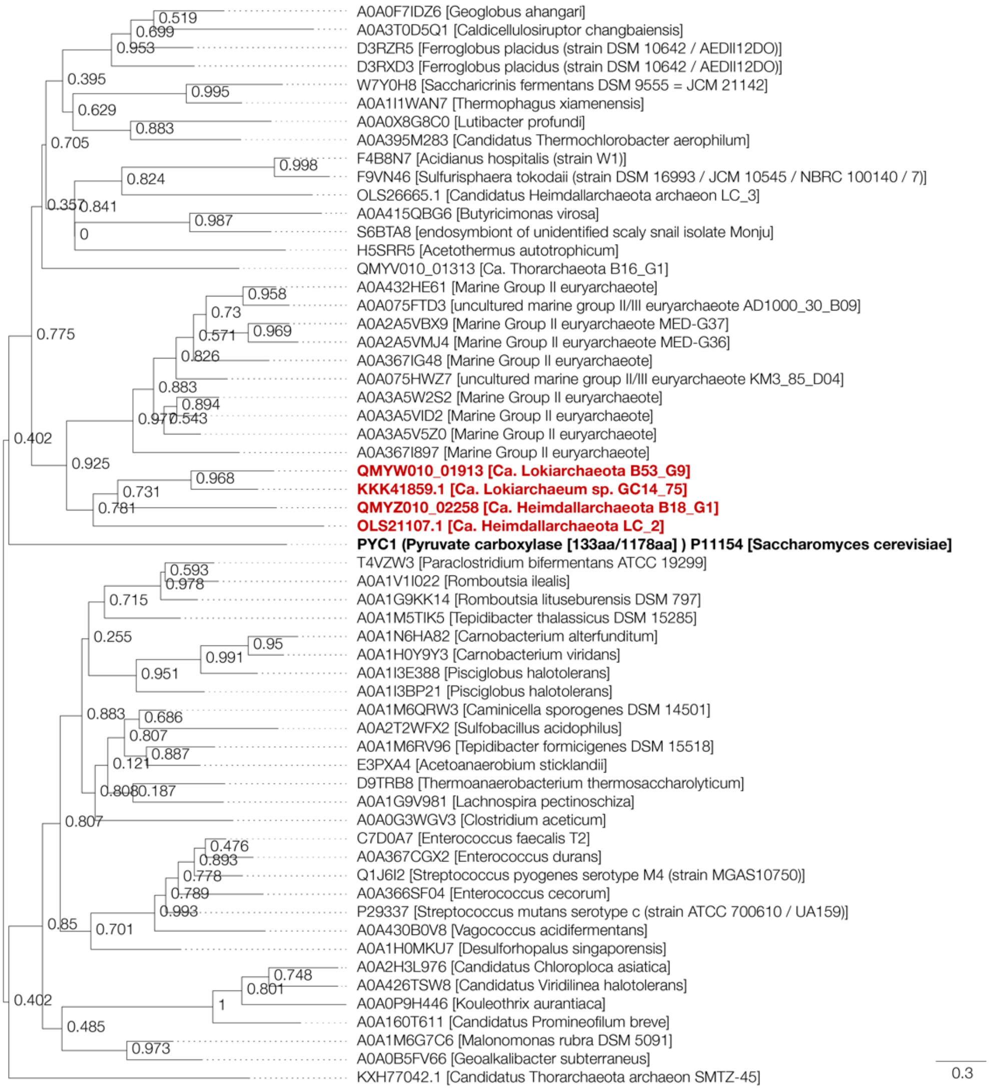

Supplementary Figure 18 | Maximum likelihood tree of Asgard archaea transcarboxylase biotin carboxyl carrier protein. See Supplementary Fig 17 caption for details. 1307 sites of the alignment were used for tree construction.

Supplementary References

1. Parkes, R. J. *et al.* A review of prokaryotic populations and processes in sub-seafloor sediments, including biosphere:geosphere interactions. *Mar. Geol.* **352,** 409–425 (2014).
2. Orcutt, B. N., Sylvan, J. B., Knab, N. J. & Edwards, K. J. Microbial ecology of the dark ocean above, at, and below the seafloor. *Microbiol. Mol. Biol. Rev.* **75,** 361–422 (2011).
3. Teske, A. P. Microbial communities of deep marine subsurface sediments: molecular and cultivation surveys. *Geomicrobiol. J.* **23,** 357–368 (2006).
4. Inagaki, F. *et al.* Biogeographical distribution and diversity of microbes in methane hydrate-bearing deep marine sediments on the Pacific Ocean Margin. *Proc. Natl. Acad. Sci. USA* **103,** 2815–2820 (2006).
5. Hinrichs, K.-U. & Inagaki, F. Downsizing the deep biosphere. *Science* **338,** 204–205 (2012).
6. Biddle, J. F. *et al.* Heterotrophic Archaea dominate sedimentary subsurface ecosystems off Peru. *Proc. Natl. Acad. Sci. USA* **103,** 3846–3851 (2006).
7. Engelen, B. & Imachi, H. *Cultivation of Subseafloor Prokaryotic Life in Developments in Marine Geology. Earth and Life Processes Discovered from Subseafloor Environment, Volume 7 –A Decade of Science Achieved by the Integrated Ocean Drilling Program (IODP)* (eds. Stein, R., Blackman, D., Inagaki, F. & Larsen, H.-L.) 197–209 (Elsevier, 2014).
8. Sass, H. & Parkes, R. J. *Sub-seafloor Sediments: An Extreme but Globally Significant Prokaryotic Habitat (Taxonomy, Diversity, Ecology) in Extremophiles Handbook* (ed. Horikoshi, K.) 1015-1041 (Springer, Tokyo, 2011).
9. Nurmiyanto, A. & Ohashi, A. Downflow hanging sponge (DHS) reactor for wastewater treatment - a short review. *MATEC Web Conf.* **280,** 05004 (2019).
10. Hatamoto, M., Okubo, T., Kubota, K. & Yamaguchi, T. Characterization of downflow hanging sponge reactors with regard to structure, process function, and microbial community compositions. *Appl. Microbiol. Biotechnol.* **102,** 10345–10352 (2018).
11. Agrawal, L. K. *et al.* Treatment of raw sewage in a temperate climate using a UASB reactor and the hanging sponge cubes process. *Water Sci. Technol.* **36,** 433–440 (1997).

12. Imachi, H. *et al.* Cultivable microbial community in 2-km-deep, 20-million-year-old subseafloor coalbeds through ~1000 days anaerobic bioreactor cultivation. *Sci. Rep.* **9,** 2305 (2019).
13. Kato, S. *et al.* Biotic manganese oxidation coupled with methane oxidation using a continuous-flow bioreactor system under marine conditions. *Water Sci. Technol.* **76,** 1781–1795 (2017).
14. Aoki, M. *et al.* A long-term cultivation of an anaerobic methane-oxidizing microbial community from deep-sea methane-seep sediment using a continuous-flow bioreactor. *PLoS ONE* **9,** e105356 (2014).
15. Imachi, H. *et al.* Cultivation of methanogenic community from subseafloor sediments using a continuous-flow bioreactor. *ISME J.* **5,** 1913–1925 (2011).
16. Nakahara, N. *et al. Aggregatilinea lenta* gen. nov., sp. nov., a slow-growing, facultatively anaerobic bacterium isolated from subseafloor sediment, and proposal of the new order *Aggregatilineales* ord. nov. within the class *Anaerolineae* of the phylum *Chloroflexi*. *Int. J. Syst. Evol. Microbiol.* **69,** 1185–1194 (2019).
17. Imachi, H. et al. *Sedimentibacter acidaminivorans* sp. nov., an anaerobic, amino acids-utilizing bacterium isolated from marine subsurface sediment. *Int. J. Syst. Evol. Microbiol.* **66,** 1293–1300 (2016).
18. Miyazaki, M. *et al. Sphaerochaeta multiformis* sp. nov., an anaerobic, psychrophilic bacterium isolated from subseafloor sediment, and emended description of the genus *Sphaerochaeta*. *Int. J. Syst. Evol. Microbiol.* **64,** 4147–4154 (2014).
19. Miyazaki, M. *et al. Spirochaeta psychrophila* sp. nov., a psychrophilic spirochaete isolated from subseafloor sediment, and emended description of the genus *Spirochaeta*. *Int. J. Syst. Evol. Microbiol.* **64,** 2798–2804 (2014).
20. Imachi, H. *et al. Pelolinea submarina* gen. nov., sp. nov., an anaerobic, filamentous bacterium of the phylum *Chloroflexi* isolated from subseafloor sediment. *Int. J. Syst. Evol. Microbiol.* **64,** 812–818 (2014).
21. Knittel, K. & Boetius, A. Anaerobic oxidation of methane: progress with an unknown process. *Annu. Rev. Microbiol.* **63,** 311–334 (2009).
22. Ruff, S. E. *et al.* Global dispersion and local diversification of the methane seep microbiome. *Proc. Natl. Acad. Sci. USA* **112,** 4015–4020 (2015).

23. Marlow, J. J. *et al.* Microbial abundance and diversity patterns associated with sediments and carbonates from the methane seep environments of Hydrate Ridge, OR. *Front. Mar. Sci.*, **1,** 44 (2014).
24. Nunoura, T. *et al.* Microbial diversity in deep-sea methane seep sediments presented by SSU rRNA gene tag sequencing. *Microbes Environ.* **27,** 382–390 (2012).
25. de Bok, F. A. M. *et al*. The first true obligately syntrophic propionate-oxidizing bacterium, *Pelotomaculum schinkii* sp. nov., co-cultured with *Methanospirillum hungatei*, and emended description of the genus *Pelotomaculum*. *Int. J. Syst. Evol. Microbiol.* **55**, 1697–1703 (2005).
26. Luo, G. *et al.* Rapid oxygenation of Earth's atmosphere 2.33 billion years ago. *Sci. Adv.* **2**, e1600134 (2016).
27. Valentine, D. L. *Thermodynamic Ecology of Hydrogen-based Syntrophy in Symbiosis. Cellular Origin, Life in Extreme Habitats and Astrobiology*, (ed. Seckbach, J.) **4**, 147–161 (Springer, Dordrecht, 2001).
28. Cypionka, H. Oxygen respiration by *Desulfovibrio* species. *Annu. Rev. Microbiol.* **54**, 827–848 (2000).
29. Kump, L. R. *et al.* Isotopic evidence for massive oxidation of organic matter following the Great Oxidation Event. *Science* **334**, 1694–1696 (2011).
30. Lyons, T. W., Reinhard, C. T. & Planavsky, N. J. The rise of oxygen in Earth's early ocean and atmosphere. *Nature* **506**, 307–315 (2014).
31. Andersson, S. G. & Kurland, C. G. Origins of mitochondria and hydrogenosomes. *Curr. Opin. Microbiol.* **2**, 535–541 (1999).
32. Fenchel, T. & Finlay, B. J. Oxygen toxicity, respiration and behavioural responses to oxygen in free-living anaerobic ciliates. *J. Gen. Microbiol.* **136**, 1953–1959 (1990).
33. Zaremba-Niedzwiedzka, K. *et al*. Asgard archaea illuminate the origin of eukaryotic cellular complexity. *Nature* **541**, 353–358 (2017).
34. Martin, W. & Müller, M. The hydrogen hypothesis for the first eukaryote. *Nature* **392**, 37–41 (1998).
35. Embley, M., Giezen, der, M. V., Horner, D. S., Dyal, P. L. & Foster, P. Mitochondria and hydrogenosomes are two forms of the same fundamental organelle. *Phil. Trans. R. Soc. Lond. B* **358**, 191–203 (2003).

36. Spang, A. *et al.* Proposal of the reverse flow model for the origin of the eukaryotic cell based on comparative analyses of Asgard archaeal metabolism. *Nat. Microbiol.* **4**, 1138–1148 (2019).
37. López-García, P. & Moreira, D. Selective forces for the origin of the eukaryotic nucleus. *BioEssays* **28,** 525–533 (2006).
38. *(Endo)symbiotic Methanogenic Archaea*. (ed. Hackstein, J. H. P.) (Springer, 2018).
39. Winkler, H. H. & Neuhaus, H. E. Non-mitochondrial ATP transport. *Trends Biochem. Sci.* **24**, 64–68 (1999).
40. Searcy, D. G. Metabolic integration during the evolutionary origin of mitochondria. *Cell Res*. **13**, 229–238 (2003).
41. Davidov, Y. & Jurkevitch, E. Predation between prokaryotes and the origin of eukaryotes. *Bioessays* **31**, 748–757 (2009).
42. Zachar, I. & Szathmáry, E. Breath-giving cooperation: critical review of origin of mitochondria hypotheses: Major unanswered questions point to the importance of early ecology. *Biology Direct* **12**, 19–26 (2017).
43. Hatamoto, M., Imachi, H., Ohashi, A. & Harada, H. Identification and cultivation of anaerobic, syntrophic long-chain fatty acid-degrading microbes from mesophilic and thermophilic methanogenic sludges. *Appl. Environ. Microbiol.* **73,** 1332–1340 (2007).
44. Daims, H., Brühl, A., Amann, R., Schleifer, K. H. & Wagner, M. The domain-specific probe EUB338 is insufficient for the detection of all *Bacteria*: development and evaluation of a more comprehensive probe set. *Syst. Appl. Microbiol.* **22,** 434–444 (1999).
45. Amann, R. I. *et al.* Combination of 16S rRNA-targeted oligonucleotide probes with flow cytometry for analyzing mixed microbial populations. *Appl. Environ. Microbiol.* **56,** 1919–1925 (1990).
46. DeLong, E. F. Archaea in coastal marine environments. *Proc. Nat.l Acad. Sci. USA* **89,** 5685–5689 (1992).
47. Weisburg, W. G., Barns, S. M., Pelletier, D. A. & Lane, D. J. 16S ribosomal DNA amplification for phylogenetic study. *J. Bacteriol.* **173,** 697–703 (1991).
48. Ludwig, W. *et al.* ARB: a software environment for sequence data. *Nucleic Acids Res.* **32,** 1363–1371 (2004).
49. Quast, C. *et al.* The SILVA ribosomal RNA gene database project: improved data

processing and web-based tools. *Nucleic Acids Res.* **41,** D590–D596 (2013).

50. Yilmaz, L. S., Parnerkar, S. & Noguera, D. R. mathFISH, a web tool that uses thermodynamics-based mathematical models for *in silico* evaluation of oligonucleotide probes for fluorescence *in situ* hybridization. *Appl. Environ. Microbiol.* **77,** 1118–1122 (2011).

51. Morono, Y., Terada, T., Kallmeyer, J. & Inagaki, F. An improved cell separation technique for marine subsurface sediments: applications for high-throughput analysis using flow cytometry and cell sorting. *Environ. Microbiol.* **15,** 2841–2849 (2013).

52. Ito, M. & Messenger, S. Isotopic imaging of refractory inclusions in meteorites with the NanoSIMS 50L. *Appl. Surf. Sci.* **255,** 1446–1450 (2008).

53. Polerecky, L. *et al.* Look@NanoSIMS - a tool for the analysis of nanoSIMS data in environmental microbiology. *Environ. Microbiol.* **14,** 1009–1023 (2012).

54. Fu, L., Niu, B., Zhu, Z., Wu, S. & Li, W. CD-HIT: accelerated for clustering the next-generation sequencing data. *Bioinformatics* **28**, 3150–3152 (2012).

55. Katoh, K. & Standley, D. M. MAFFT multiple sequence alignment software version 7: improvements in performance and usability. *Mol. Bio.l Evol.* **30,** 772–780 (2013).

56. Capella-Gutiérrez, S., Silla-Martínez, J. M. & Gabaldón, T. trimAl: a tool for automated alignment trimming in large-scale phylogenetic analyses. *Bioinformatics* **25,** 1972–1973 (2009).

57. Price, M. N., Dehal, P. S. & Arkin, A. P. FastTree 2-approximately maximum-likelihood trees for large alignments. *PLoS ONE* **5,** e9490 (2010).

58. Kozlov, A. M., Darriba, D., Flouri, T., Morel, B. & Stamatakis, A. RAxML-NG: A fast, scalable, and user-friendly tool for maximum likelihood phylogenetic inference. *Bioinformatics* **35**, 4453–4455 (2019).
