# Supplementary material for: Isolation of an archaeon at the prokaryote–eukaryote interface
Source: Nature. 2020 Jan 15;577(7791):519–25. doi: 10.1038/s41586-019-1916-6 (PMC7015854; doi:10.1038/s41586-019-1916-6)
Supplement: Supplementary file 2 — Reporting Summary [file 41586_2019_1916_MOESM2_ESM.pdf]

nature research

Corresponding author(s): Hiroyuki Imachi
Masaru K. Nobu

Last updated by author(s): Nov 26, 2019

Reporting Summary

Nature Research wishes to improve the reproducibility of the work that we publish. This form provides structure for consistency and transparency in reporting. For further information on Nature Research policies, see Authors & Referees and the Editorial Policy Checklist .

Statistics

For all statistical analyses, confirm that the following items are present in the figure legend, table legend, main text, or Methods section.

n/a	Confirmed	
☐	☒	The exact sample size (*n*) for each experimental group/condition, given as a discrete number and unit of measurement
☐	☒	A statement on whether measurements were taken from distinct samples or whether the same sample was measured repeatedly
☒	☐	The statistical test(s) used AND whether they are one- or two-sided<br>*Only common tests should be described solely by name; describe more complex techniques in the Methods section.*
☒	☐	A description of all covariates tested
☒	☐	A description of any assumptions or corrections, such as tests of normality and adjustment for multiple comparisons
☒	☐	A full description of the statistical parameters including central tendency (e.g. means) or other basic estimates (e.g. regression coefficient) AND variation (e.g. standard deviation) or associated estimates of uncertainty (e.g. confidence intervals)
☒	☐	For null hypothesis testing, the test statistic (e.g. *F*, *t*, *r*) with confidence intervals, effect sizes, degrees of freedom and *P* value noted<br>*Give P values as exact values whenever suitable.*
☒	☐	For Bayesian analysis, information on the choice of priors and Markov chain Monte Carlo settings
☒	☐	For hierarchical and complex designs, identification of the appropriate level for tests and full reporting of outcomes
☒	☐	Estimates of effect sizes (e.g. Cohen's *d*, Pearson's *r*), indicating how they were calculated

Our web collection on statistics for biologists contains articles on many of the points above.

Software and code

Policy information about availability of computer code

Data collection	MiSeq control software v3.1 for sequencing; StepOne Software v2.3 for qPCR; Nikon NIS-Elements software for FISH imaging
Data analysis	IDL based NASA JSC imaging software, OpenMIMS (https://github.com/BWHCNI/OpenMIMS), and Look@NanoSIMS for NanoSIMS; IMOD for tomogram reconstruction; Trimmomatic v0.33 and NextClip v1.3.1 for trimming; SPAdes v3.1.1 for genome assembly; MyCC (2015/07/10) for genome binning; SSPACE v3.0 for scaffolding; Prokka v1.12 for genome annotation; SignalP v4.1 for signal peptide prediction; MAFFT v7 for gene sequence alignment; RAxML-NG v0.8.0 for maximum likelihood tree construction; trimAl v1.2 for alignment trimming; CD-HIT v.4.8.1 for gene clustering; MrBayes 3.2.7a for Bayesian phylogenetic tree calculation; PhyML 3.3 for maximum likelihood tree construction; MUSCLE v3.8.31 for sequence alignment.

For manuscripts utilizing custom algorithms or software that are central to the research but not yet described in published literature, software must be made available to editors/reviewers. We strongly encourage code deposition in a community repository (e.g. GitHub). See the Nature Research guidelines for submitting code & software for further information.

Data

Policy information about availability of data

All manuscripts must include a data availability statement. This statement should provide the following information, where applicable:

- Accession codes, unique identifiers, or web links for publicly available datasets
- A list of figures that have associated raw data
- A description of any restrictions on data availability

Genomes for Ca. Prometheoarchaeum syntrophicum MK-D1, Halodesulfovibrio sp. MK-HDV, and Methanogenium sp. MK-MG are available under Genbank BioProjects PRJNA557562, PRJNA557563, and PRJNA557565 respectively. The iTAG sequence data was deposited in Bioproject PRJDB8518 with the accession numbers DRR184081–DRR184101. The 16S rRNA gene sequences of MK-D1, Halodesulfovibrio, Methanogenium and clones obtained from primary enrichment culture were deposited in the DDBJ/EMBL/GenBank database under accession numbers LC490619–LC490624.

Field-specific reporting

Please select the one below that is the best fit for your research. If you are not sure, read the appropriate sections before making your selection.

- [x] Life sciences
- [ ] Behavioural & social sciences
- [ ] Ecological, evolutionary & environmental sciences

For a reference copy of the document with all sections, see nature.com/documents/nr-reporting-summary-flat.pdf

Life sciences study design

All studies must disclose on these points even when the disclosure is negative.

Sample size	Sample size-based calculations not relevant to analyses in this study
Data exclusions	No data were excluded from the analysis
Replication	Culture experiments were performed in duplicate or triplicate. RNA-based experiments were performed without replicates due to challenges in cultivation (i.e., extremely low growth rates and culture densities)
Randomization	Randomization not relevant to data collection/analyses in this study as the study does not involve participant groups. Each experiment included controls.
Blinding	Blinding not relevant to data collection performed in this study as blinding is not required and was not possible for cultivation-based experiments as the investigators must verify the control and non-control groups for each experiment.

Reporting for specific materials, systems and methods

We require information from authors about some types of materials, experimental systems and methods used in many studies. Here, indicate whether each material, system or method listed is relevant to your study. If you are not sure if a list item applies to your research, read the appropriate section before selecting a response.

Materials & experimental systems

n/a	Involved in the study	
x		Antibodies
x		Eukaryotic cell lines
x		Palaeontology
x		Animals and other organisms
x		Human research participants
x		Clinical data

Methods

n/a	Involved in the study	
x		ChIP-seq
x		Flow cytometry
x		MRI-based neuroimaging
